# A Non-Targeted LC-MS Profiling Reveals Elevated Levels of Carnitine Precursors and Trimethylated Compounds in the Cord Plasma of Pre-Eclamptic Infants

**DOI:** 10.1038/s41598-018-32804-5

**Published:** 2018-10-02

**Authors:** Tiina Jääskeläinen, Olli Kärkkäinen, Jenna Jokkala, Kaisa Litonius, Seppo Heinonen, Seppo Auriola, Marko Lehtonen, Kati Hanhineva, Hannele Laivuori, FINNPEC, Eero Kajantie, Juha Kere, Katja Kivinen, Anneli Pouta

**Affiliations:** 1 Medical and Clinical Genetics, University of Helsinki and Helsinki University Hospital, Helsinki, Finland; 2 Institute of Public Health and Clinical Nutrition, University of Eastern Finland, Kuopio, Finland; 3 Obstetrics and Gynecology, University of Helsinki and Helsinki University Hospital, Helsinki, Finland; 4 School of Pharmacy, University of Eastern Finland, Kuopio, Finland; 5 Institute for Molecular Medicine Finland (FIMM), Helsinki Institute of Life Science, University of Helsinki, Helsinki, Finland; 6 Department of Obstetrics and Gynecology, Tampere University Hospital and University of Tampere, Faculty of Medicine and Life Sciences, Tampere, Finland; 7 Chronic Disease Prevention Unit, National Institute for Health and Welfare, Helsinki, Finland; 8 Children’s Hospital, Helsinki University Hospital and University of Helsinki, Helsinki, Finland; 9 PEDEGO Research Unit, MRC Oulu, Oulu University Hospital and University of Oulu, Oulu, Finland; 10 Department of Biosciences and Nutrition, and Science for Life Laboratory, Karolinska Institutet, Stockholm, Sweden; 11 Molecular Neurology Research Program, University of Helsinki, Helsinki, Finland; 12 Folkhälsan Institute of Genetics, Helsinki, Finland; 13 Division of Cardiovascular Medicine, University of Cambridge, Cambridge, UK; 14 Department of Government Services, National Institute for Health and Welfare, Helsinki, Finland

## Abstract

Preeclampsia (PE) is a complex pregnancy disorder. It is not extensively known how the metabolic alterations of PE women contribute to the metabolism of newborn. We applied liquid chromatography-mass spectrometry (LC-MS) based non-targeted metabolomics to determine whether the metabolic profile of plasma from umbilical cord differs between infants born to PE and non-PE pregnancies in the FINNPEC study. Cord plasma was available from 42 newborns born from PE and 53 from non-PE pregnancies. 133 molecular features differed between PE and non-PE newborns after correction for multiple testing. Decreased levels of 4-pyridoxic acid were observed in the cord plasma samples of PE newborns when compared to non-PE newborns. Compounds representing following areas of metabolism were increased in the cord plasma of PE newborns: urea and creatine metabolism; carnitine biosynthesis and acylcarnitines; putrescine metabolites; tryptophan metabolism and phosphatidylcholines. To our knowledge, this study is the first one to apply LC-MS based metabolomics in cord plasma of PE newborns. We demonstrate that this strategy provides a global picture of the widespread metabolic alterations associated with PE and particularly the elevated levels of carnitine precursors and trimethylated compounds appear to be associated with PE at birth.

## Introduction

Pre-eclampsia (PE) is a complex metabolic and vascular pregnancy disorder characterized by new-onset hypertension and proteinuria after 20 weeks of gestation, or new-onset PE-associated signs in the absence of proteinuria^1^. The etiology of PE remains largely unclear, however, defective placentation is frequently present. The pathology is also characterized by endothelial dysfunction, metabolic changes, angiogenic imbalance, hypoxia, and aberrant immune system^2,3^. The imminent effects on the fetus include prematurity (due to indicated preterm deliveries) and fetal growth impairment^4^. The metabolic alterations of PE women may also contribute to the metabolism of fetus^5^. However, it is not currently well understood to what extent the metabolic changes observed in PE women also affect the newborn.

Metabolomics allows for the concomitant measurement of a wide spectrum of low molecular weight metabolites resulting from endogenous metabolism, dietary intake and gut microbial activity^6,7^. It has been shown to generate new insights when investigating physiological status due to disease or treatment and thus, may also provide new insights into the pathogenesis of PE. Previously, differential metabolic profiles have been characterized in PE across a variety of analytical platforms, in different maternal biospecimens (placenta, blood, serum, urine and amniotic fluid^8–13^. For instance, changes in lipid and amino acid metabolism and dysregulation of mitochondria have been observed. However, relatively scarce data are available on newborns.

In this study, we have taken a non-targeted approach to identify a metabolic signature in cord plasma of infants born to PE and non-PE pregnancies in the FINNPEC (Finnish Genetics of Preeclampsia Consortium) study in order to elucidate the metabolic changes related to PE.

## Methods

### Study cohort

The FINNPEC is a cross-sectional case-control multicentre study with a nationwide clinical and DNA database on PE and non-PE women, including their partners and newborns. Data of the prospective arm was assembled in Finland between 2008 and 2011. Details of the study design, methods and procedures have been described elsewhere^14^. All participants provided written informed consent, and the FINNPEC study protocol was approved by the coordinating Ethics Committee of the Hospital District of Helsinki and Uusimaa. All experiments were performed in accordance with relevant guidelines and regulations.

PE was defined as hypertension and proteinuria occurring after 20 weeks gestation according to the American College of Obstetricians and Gynecologists (ACOG) 2002 criteria. Cord blood samples were collected after delivery from a subcohort from the Hospital District of Helsinki and Uusimaa only (n = 486 for the PE newborns and n = 526 for the non-PE newborns). For the current LC-MS metabolite profiling analysis, we selected samples of newborns whose mothers were non-smoking and from whom we had maternal first and third trimester serum samples available (n = 42 for PE newborns and n = 53 for the non-PE newborns, Table 1). After delivery, the umbilical cord was double clamped and the cord blood sample was collected in the 10 ml EDTA tubes, centrifuged, the plasma was removed and stored at −80 °C.

**Table 1:** Maternal and fetal characteristics of the study groups.

Maternal characteristics	Pre-eclampsia (n = 42)	Control (n = 53)	*p* ^unadj.^
Age at delivery, year	31.3 ± 4.7 (mean ± SD)	30.4 ± 4.6	0.371
Gravidity	1.9 ± 1.2	2.1 ± 1.3	0.407^a^
Parity	0.3 ± 0.5	0.6 ± 0.7	**0.029** ^**a**^
Nulliparous	30 (71.4%)	27 (50.9%)	**0.043**
**Onset of pre-eclampsia (based of delivery)**			
Early (≤34 + 0 weeks of gestation)	7 (16.7%)	—	—
Late (>34 + 0 weeks of gestation)	35 (83.3%)	—	—
History of pre-eclampsia	6 (14.3%)	0 (0%)	**0.006**
Weight, kg (self-reported, pre-pregnancy)	68.8 ± 10.6	70.5 ± 8.9	0.337
Height, m	1.66 ± 0.07	1.67 ± 0.06	0.389
BMI, kg/m^2^ (self-reported, pre-pregnancy)	25.1 ± 4.0	25.2 ± 2.5	0.644
Systolic blood pressure at first antenatal visit, mm Hg	122 ± 12	115 ± 9	**0.003**
Diastolic blood pressure at first antenatal visit, mm Hg	79 ± 10	72 ± 7	**<0.001**
Highest systolic blood pressure, mm Hg	171 ± 17	124 ± 9	**<0.001** ^**a**^
Highest diastolic blood pressure, mm Hg	110 ± 9	83 ± 6	**<0.001** ^**a**^
Proteinuria (maximum), g/24 hour	4.3 ± 4.2	—	—
Chronic hypertension	8 (19.0%)	0 (0%)	**0.001**
Gestational hypertension	—	0 (0%)	—
Gestational diabetes mellitus	6 (14.3%)	0 (0%)	**0.006**
Pregestational diabetes mellitus	4 (9.5%)	0 (0%)	**0.035**
Type 1 diabetes	3 (7.1%)	0 (0%)	0.083
Type 2 diabetes	1 (2.4%)	0 (0%)	0.442
Mode of delivery			
Vaginal	26 (61.9%)	46 (86.7%)	**0.005**
Caesarean section	16 (38.1%)	7 (13.2%)	**0.005**
Prenatal betamethasone treatment	10 (23.8%)	0 (0%)	**<0.001**
**Fetal characteristics**			
Birth weight, g	2866 ± 824	3596 ± 423	**<0.001** ^**a**^
Relative birth weight, SD	−0.8 ± 1.2	0.0 ± 0.8	**<0.001**
SGA	3 (7.1%)	0 (0%)	0.083
Gestational weeks	37.0 ± 3.2	39.7 ± 1.3	**<0.001** ^**a**^
Sex			0.614
Male	20 (47.6%)	28 (52.8%)	
Female	22 (52.4%)	25 (47.2%)	

Chronic hypertension was defined as systolic blood pressure ≥140 mm Hg and/or diastolic blood pressure ≥90 mm Hg detected before 20 weeks of gestation. Gestational hypertension was defined as blood pressure ≥140/90 without proteinuria. SGA = small-for-gestational age. ^a^Non-parametric test was used.

### Non-targeted LC-MS metabolite profiling and identification

The sample preparation, instrument parameters and processing of data were performed in the LC–MS Metabolomics Center at Biocenter Kuopio (University of Eastern Finland). An aliquot (100 μL) of stored (−80 °C) plasma samples was mixed with 400 μL of acetonitrile (ACN; VWR International, Leuven, Belgium), and mixed in vortex at maximum speed 15 s, incubated on ice bath for 15 min to precipitate the proteins, and centrifuged at 16 000 × g for 10 min to collect the supernatant. The supernatant was filtered through 0.2 μm PTFE filters. Aliquots of 2 μL were taken from at least half of the plasma samples, mixed together in one tube, and used as the quality control sample in the analysis. Additionally a solvent blank was prepared in the same manner.

The samples were analyzed by the UHPLC-qTOF-MS system (Agilent Technologies, Waldbronn, Karlsruhe, Germany) that consisted of a 1290 LC system, a Jetstream electrospray ionization (ESI) source, and a 6540 UHD accurate-mass qTOF spectrometer. The samples were analyzed using two different chromatographic techniques, i.e. reversed phase (RP) and hydrophilic interaction (Hilic) chromatography. Data were acquired in both positive (+) and negative (−) polarity. The sample tray was kept at 4 °C during the analysis. The data acquisition software was MassHunter Acquisition B.04.00 (Agilent Technologies). The quality control and the blank samples were injected after every 12 samples and also in the beginning of the analysis. The sample order of the analysis was randomized. Details on the technical procedures and parameters have been described earlier by Pekkinen *et al*.^15^.

Data were collected with “Find by Molecular Feature” algorithm in MassHunter Qualitative Analysis B.07.00 software (Agilent Technologies, USA). The extraction algorithm was set to collect peaks with threshold at 200 counts for Hilic and 150 for RP chromatography, and the allowed ion species were [M + H]+, [M + Na]+, [M + K]+, [M + NH4]+, and [2M + H]+in ESI(+), and [M−H]−, [M + Cl]−, [M + HCOO]−, [M + CF3COO]−, and [2M − H]- in ESI(−). Only signals over compound height threshold of 3000 counts containing at least two ions were included in the compound list. Peak spacing tolerance for isotope grouping was 0.0025 m/z plus 7 ppm, with isotope model for common organic molecules. Data files (.cef-format) were exported to Mass Profiler Professional (Agilent Technologies) for peak alignment. After the first initial alignment, the data were combined in one.cef file, against which the original raw data was reanalyzed. For this recursive analysis, compound mass tolerance was ±15 ppm, retention time ±0.150 min and symmetric expansion value for chromatograms ±35.0 ppm. Resulting compounds were re-exported to Mass Profiler Professional software for peak alignment and data cleanup.

### Statistical analyses

Statistical analysis of the metabolite profiling data was performed using univariate and multivariate analysis methods. For univariate analysis, we calculated *p*-values (Student’s t-test, α level = 0.05) and effects sizes (Cohen’s d = (mean(cases)-mean(controls)/((SD(cases) + SD(controls))/2)) for comparisons between PE cases and non-PE controls for each molecular feature. Principal component analysis (PCA) was used to analyze overall variance within the samples. Because of the correlative nature of molecular features in a metabolite profile analysis, we used the number of principal components needed to explain 95% of variance in the data to adjust the t-test α level for multiple test correction in the metabolic profiling analysis (Bonferroni’s method). Furthermore, we used partial-least-squares discriminant-analysis (PLS-DA) to find out the variables explaining most variation between PE and non-PE subjects. For the PLS-DA, variables were normalized by standard deviation (z-score) and mean centered before analysis. Variable importance to the projection (VIP) values are reported for each variable. We used the Mass Profiler Professional (MPP) software (Agilent Technologies, version 13) to calculate p-values, Microsoft Excel 2016 to calculate Cohen’s d values, and SIMCA (Umetrics, version 14.0.0) to perform multivariate analyses. Moreover, we used MS-DIAL ver.2.52^16^ and MassHunter Qualitative Analysis B.07.00 software (Agilent Technologies, USA) for metabolite identification against MSMS spectra found in public and in-house standard libraries. In-house database was generated by analyzing commercial and synthetized chemical standards in the same analytical conditions using the same instrumentation, and comprised of 634 compounds. For public databases, we used Metlin (https://metlin.scripps.edu/, accessed 23.3.2017) and the internal database found in MS-DIAL (version 2.52).

Figures were made with Prism (GraphPad Software Inc., version 5.03) and SIMCA (Umetrics, version 14.0.0).

## Results

Maternal and fetal characteristics are presented in the Table 1. There were no differences in maternal age or body mass index (BMI) between the PE and non-PE groups. All women were non-smoking. PE women had elevated diastolic and systolic blood pressure and proteinuria as expected according to the diagnostic criteria used for PE. The proportion of nulliparous women was higher in the PE group compared with the non-PE group. PE women also suffered more from pre-existing diseases (e.g. chronic hypertension, pregestational diabetes and gestational diabetes) and had more frequently history of PE compared to non-PE women. PE women had more caesarean deliveries than non-PE women. Ten out of 42 PE women received prenatal betamethasone treatment for fetal lung maturation. The newborns of PE pregnancies were born earlier and had smaller absolute and relative birth weight (Table 1). There was no difference in the sex distribution of newborns.

**Figure 1:**
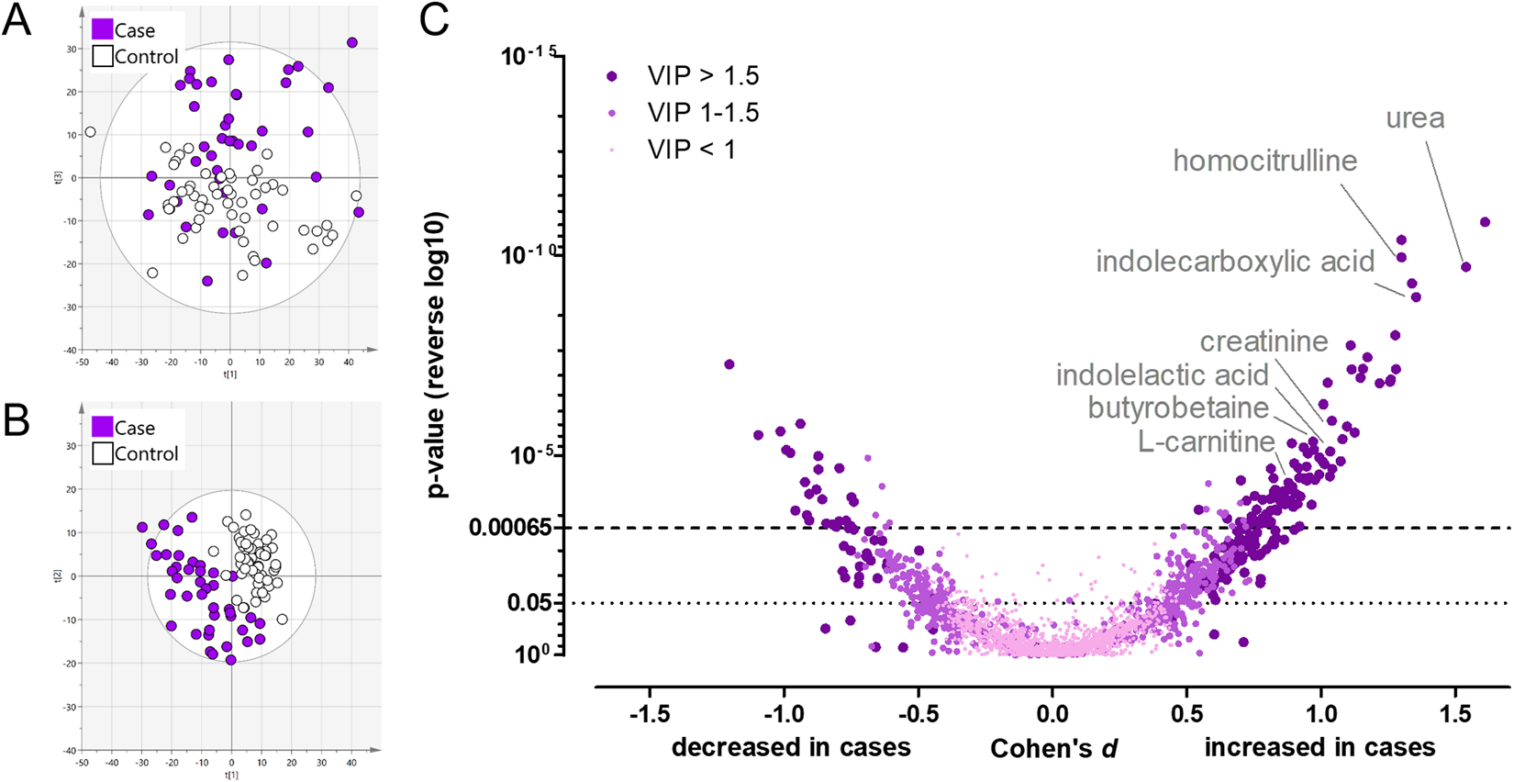
Overview of the metabolite profiling results from the cord plasma samples.Total of 2401 molecular features were observed from the cord plasma samples. Panel **A** is showing the first and third component of a principal component analysis (PCA). Total of 77 component were needed to cumulatively explain 95% of the variance in the data. Panel **B** shows first two components of a partial-least-squares discriminant-analysis (PLS-DA). In the PLS-DA model (**B**), five components cumulatively explain 96% of the variance in the data between the groups. These five components are able to predict 57% of the data (leave-one-out cross-validation). Panel **C** shows the p-values (Student’s t-test), Cohen’s d effect sizes and VIP-values (from PLS-DA) of all molecular features. Total of 133 molecular features were significantly altered between newborns of PE and non-PE women after correction for multiple testing (adjusted α level = 0.00065, Bonferroni’s correction).

In the metabolite profiling analysis we observed a total of 2401 molecular features of which 538 had *p*-values below 0.05 **(**Supplementary Table 1**)**. Urea, indolecarboxylic acid, 2PY, homocitrulline, indolelactic acid, 1-methylhistidine, creatinine, PC 16:0/16:0, PC 16:0/18:1, and diacetylspermidine were metabolites contributing most to the separation of the groups in the PLS-DA (i.e. had the highest VIP-values, Supplementary Table 1). The PLS-DA model had five components with cumulative R2Y of 0.96 (percent of variation explained by the model) and Q2 of 0.57 (percent of variation predicted by the model according to cross validation). In the PCA analysis, total of 77 compounds were needed to explain 95% of the overall variance in the data. This result was used to adjust the t-test α-level to 0.00065 to account for multiple testing (Bonferroni’s method). This resulted in total of 133 molecular features which were statistically significantly altered between the umbilical cord plasma of newborns of PE and non-PE pregnancies (Fig. 1). The analyses were also re-performed excluding mothers suffering from gestational diabetes but only minor changes were observed (analyses not shown).

**Figure 2:**
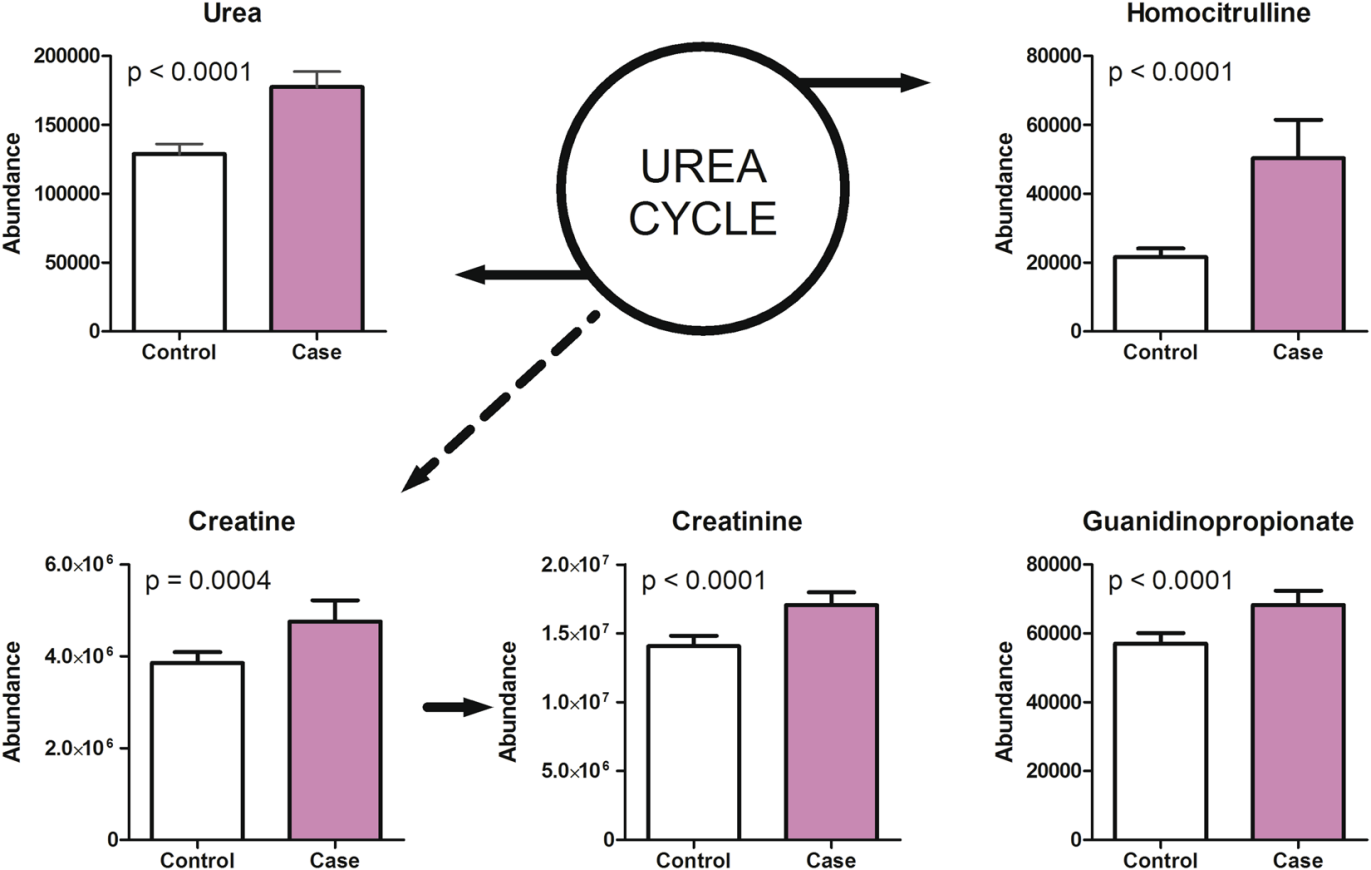
Metabolites associated with urea cycle and creatine metabolism were increased in cord plasma samples of preeclampsia pregnancies. Urea, creatine, creatinine, homocitrulline and guanidinopropionate levels were significantly increased in the umbilical cord plasma samples of preeclampsia cases when compared to controls. Mean and 95% confidence intervals are show for the groups as well as p-values from t-test.

**Figure 3:**
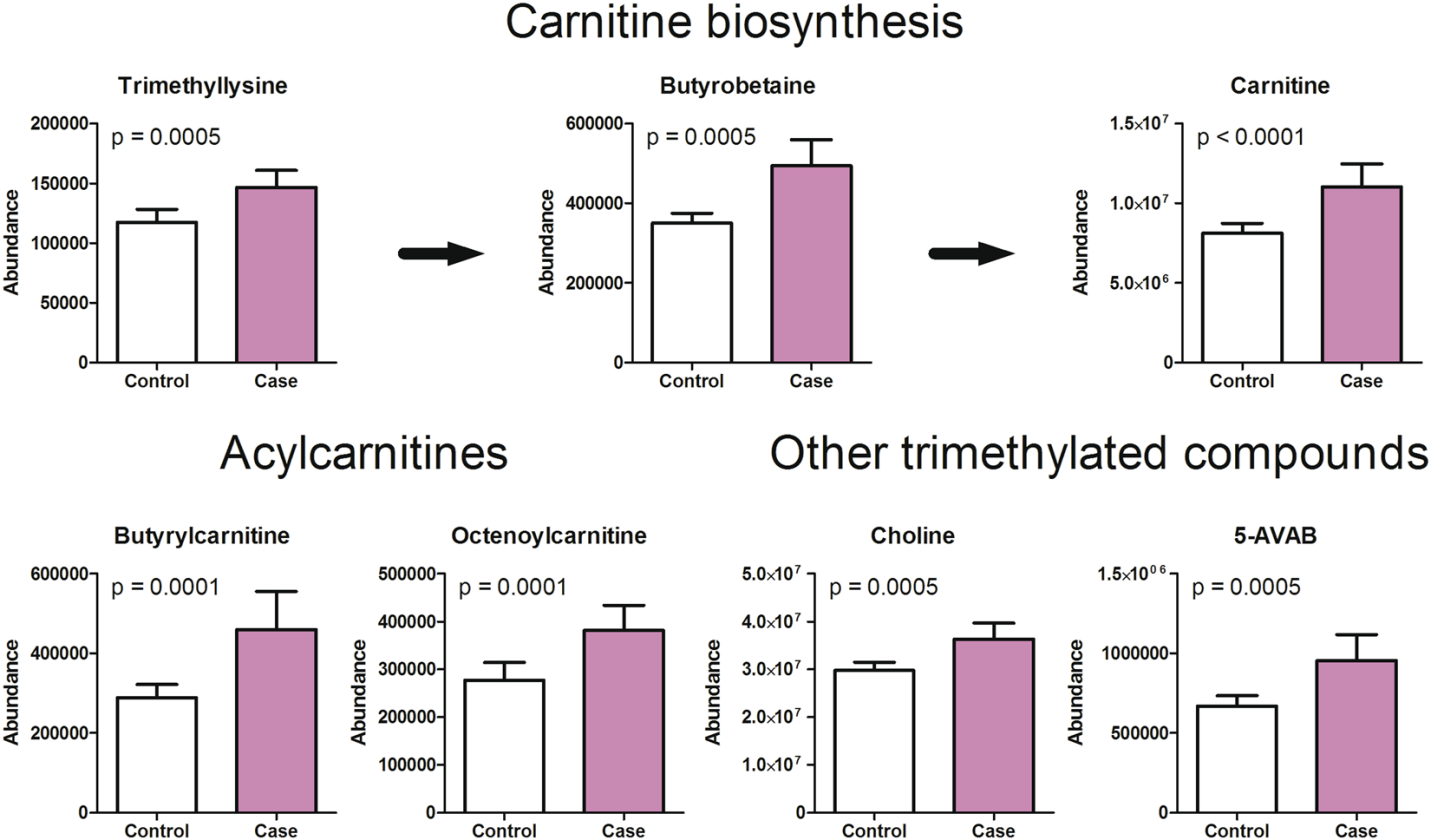
Trimethylated metabolites were increased in cord plasma samples of preeclampsia pregnancies. Increased levels of carnitine, trimethyllysine, butyrobetaine, butyrylcarnitine, octenoylcarnitine, choline and 5-aminovaleric acid betaine (5-AVAB) were observed in the umbilical cord plasma samples of preeclampsia newborns when compared to controls. Trimethyllysine and butyrobetaine are precursors of carnitine. Mean and 95% confidence intervals are show for the groups as well as p-values from t-test.

Compounds related to urea and creatine metabolism (urea, creatine, creatinine, homocitrulline and guanidinopropionate) were increased in the cord plasma of newborns of PE pregnancies compared to non-PE cord plasma (Fig. 2). Furthermore, metabolites of carnitine biosynthesis and acylcarnitines were increased [carnitine, trimethyllysine, gamma-butyrobetaine, butyrylcarnitine, octenoylcarnitine and 5-Amino valeric acid betaine (5-AVAB)] in the cord plasma of newborns of PE pregnancies (Fig. 3).

The newborns of PE pregnancies also showed increased levels of N-methyl-2-pyridone-5-carboxamide (2PY). Moreover, two putrescine metabolites, acetylputrescine and 4-acetamidobutanoate, were increased in the cord plasma of newborns of PE pregnancies. Similarly two metabolites, indolecarboxylic acid and indolelactic acid, associated with tryptophan metabolism were increased in the PE newborns. Furthermore, levels of phosphatidylchlolines (PCs) PC(16:1/18:1), PC(16:0/16:0), and PC(16:0/18:1) were increased in the PE newborns compared to non-PE newborns. Decreased levels of 4-pyridoxic acid was observed in the cord plasma samples of PE newborns.

**Figure 4:**
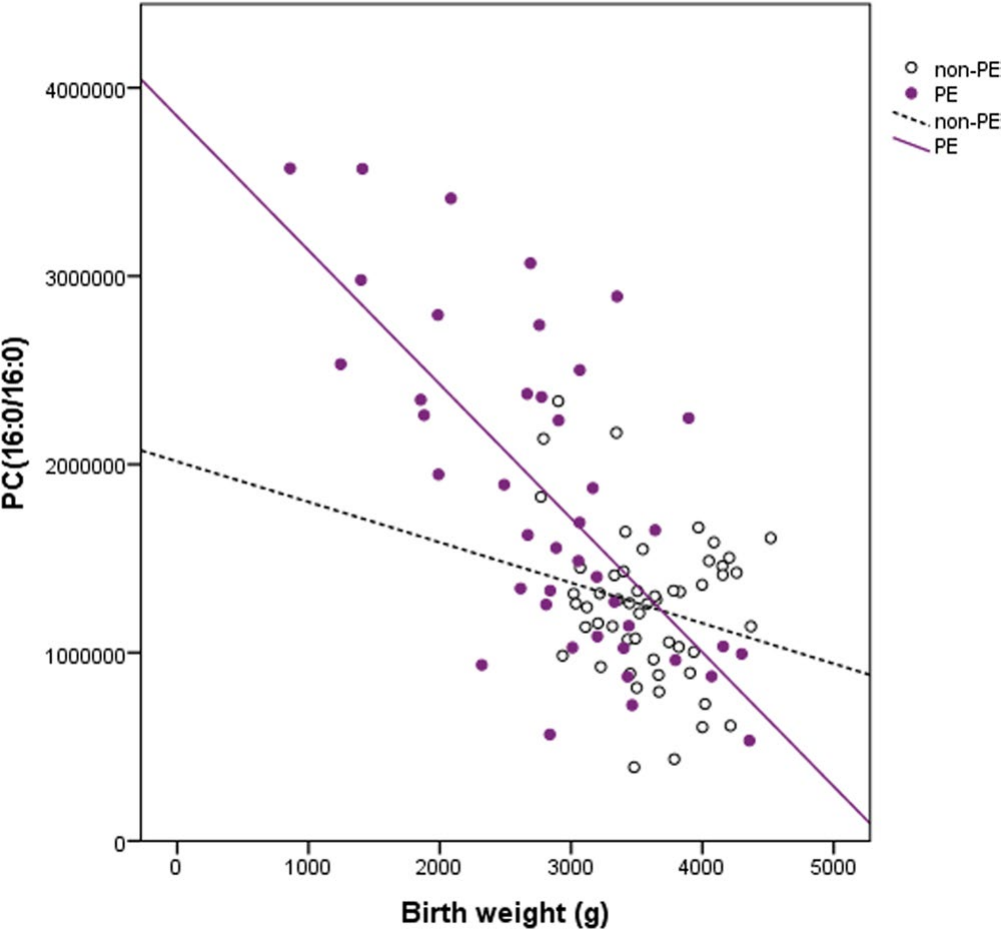
Correlation of phosphatidylcholine (PC) 16:0/16:0 and birth weight. Data from non-pre-eclamptic (open circles) and pre-eclamptic (closed circles) newborns.

The correlations between birth weight, SD, gestational weeks and metabolites are presented in Table 2. The strongest negative associations among PE newborns were found between birth weight, gestational weeks and PCs. The correlation between PC(16:0/16:0) and birth weight is illustrated in Fig. 4. Table 2

**Table 2:** Pearson’s correlation coefficients (r) of metabolites and birth weight (BWT), relative weight (SD) and gestational weeks (GWKs) in all study subjects, in PE and non-PE groups. SD= birth weight and height converted to standard deviation scores relative to the Finnish references^58^; NA= data not available.

Metabolite		BWT		SD		GWKs		Maternal-fetal transfer based on human studies (yes/no)	Previous literature on cord blood/plasma in PE
		r	p	r	p	r	p		
**UREA CYCLE AND CREATINE BIOSYNTHESIS**									
Urea	**All**	−0.444	**<0.001**	−0.29	**0.004**	−0.419	**<0.001**	Yes^51^	↑ in umbilical cord blood of severe PE cases^33^ ↑ in the amniotic fluid^32^
	**PE**	−0.428	**0.005**	−0.218	0.172	−0.426	**0.005**		
	**Non**-**PE**	0.256	0.064	0.099	0.480	0.469	**<0.001**		
Creatine	**All**	−0.293	**0.004**	−0.146	0.159	−0.331	**0.001**	Yes^52^	NA
	**PE**	−0.169	0.286	0.041	0.796	−0.247	0.115		
	**Non**-**PE**	−0.063	0.652	−0.118	0.400	−0.002	0.991		
Creatinine	**All**	−0.26	**0.011**	−0.193	0.061	−0.193	0.061	NA	↑ in the amniotic fluid^32^
	**PE**	−0.097	0.541	0.045	0.779	−0.180	0.254		
	**Non**-**PE**	−0.058	0.679	−0.112	0.423	0.531	**<0.001**		
Homocitrulline	**All**	0.295	**0.004**	−0.138	0.182	−0.339	**0.001**	NA	NA
	**PE**	−0.087	0.582	0.067	0.674	−0.141	0.374		
	**Non**-**PE**	0.123	0.382	0.063	0.654	0.040	0.774		
Guanidinopropionate	**All**	−0.44	**0.001**	−0.205	**0.046**	−0.527	**<0.001**	NA	NA
	**PE**	−0.507	**0.001**	−0.107	0.501	−0.638	**<0.001**		
	**Non**-**PE**	0.098	0.485	0.008	0.957	0.085	0.543		
**CARNITINE, CHOLINE AND BETAINE METABOLISM**									
Trimethyllysine	**All**	−0.221	**0.031**	−0.133	0.200	−0.270	**0.008**	NA	NA
	**PE**	−0.017	0.917	0.149	0.345	−0.128	0.418		
	**Non**-**PE**	−0.169	0.228	−0.217	0.119	−0.158	0.259		
γ-butyrobetaine	**All**	−0.413	**<0.001**	−0.183	0.076	−0.508	**<0.001**	NA	NA
	**PE**	−0.295	0.058	0.021	0.896	−0.555	**<0.001**		
	**Non**-**PE**	−0.124	0.375	−0.173	0.217	0.329	**0.016**		
Carnitine	**All**	−0.434	**< 0.001**	−0.211	**0.04**	−0.501	**<0.001**	Yes^22,53,54^	↑ in umbilical vein endothelial cells^21^
	**PE**	−0.325	**0.036**	0.019	0.907	−0.476	**0.001**		
	**Non**-**PE**	−0.212	0.128	−0.297	**0.031**	−0.017	0.902		
Butyrylcarnitine	**All**	−0.516	**<0.001**	−0.286	**0.005**	−0.508	**<0.001**	Yes^54,55^	↑ in umbilical vein endothelial cells^21^
	**PE**	−0.532	**<0.001**	−0.215	0.171	−0.555	**<0.001**		
	**Non**-**PE**	0.030	0.829	−0.092	0.511	0.329	**0.016**		
Octenoylcarnitine	**All**	−0.334	**0.001**	−0.284	**0.005**	−0.244	**0.017**	Yes^54,55^	↑ in umbilical vein endothelial cells^21^
	**PE**	−0.322	**0.038**	−0.229	0.145	−0.297	0.056		
	**Non**-**PE**	0.006	0.964	−0.125	0.374	0.374	**0.006**		
Choline	**All**	−0.341	**0.001**	−0.257	**0.012**	−0.298	**0.003**	Yes^56^	NA
	**PE**	−0.313	**0.044**	−0.202	0.199	−0.282	0.071		
	**Non**-**PE**	0.103	0.463	−0.036	0.796	0.294	**0.032**		
5-AVAB	**All**	−0.200	0.052	−0.036	0.732	−0.264	**0.010**	NA	NA
	**PE**	−0.053	0.737	0.185	0.240	−0.168	0.287		
	**Non**-**PE**	0.048	0.734	−0.066	0.637	0.124	0.374		
**VITAMIN B6 METABOLISM**									
4-pyridoxic acid	**All**	0.266	**0.009**	0.217	**0.035**	0.309	**0.002**	NA	NA
	**PE**	0.345	**0.025**	0.316	**0.041**	0.265	0.090		
	**Non**-**PE**	−0.014	0.922	0.008	0.955	0.179	0.199		
**NICOTINAMIDE METABOLISM**									
2PY (N-methyl-2-pyridone-5-carboxamide)	**All**	−0.426	**<0.001**	−0.28	**0.006**	−0.408	**<0.001**	NA	NA
	**PE**	−0.402	**0.008**	−0.187	0.235	−0.408	**0.007**		
	**Non**-**PE**	0.125	0.372	0.008	0.954	0.286	**0.038**		
**POLYAMINE METABOLISM**									
Acetylputrescine	**All**	−0.419	**<0.001**	−0.109	0.291	−0.577	**<0.001**	NA	NA
	**PE**	−0.426	**0.005**	0.022	0.890	−0.627	**<0.001**		
	**Non-PE**	0.069	0.626	0.059	0.673	−0.051	0.717		
4-acetamido-butanoate	**All**	−0.088	0.397	0.068	0.510	−0.224	**0.029**	NA	NA
	**PE**	0.171	0.280	0.366	**0.017**	−0.027	0.867		
	**Non-PE**	0.025	0.858	−0.021	0.883	−0.070	0.619		
**TRYPTOPHAN METABOLISM**									
Indolecarboxylic acid	**All**	−0.385	**<0.001**	−0.168	0.104	−0.471	**<0.001**	Yes^57^	NA
	**PE**	−0.241	0.124	0.065	0.682	−0.381	**0.013**		
	**Non-PE**	0.103	0.464	0.019	0.890	0.091	0.515		
Indolelactic acid	**All**	−0.293	**0.004**	−0.169	0.102	−0.306	**0.003**	Yes^57^	NA
	**PE**	−0.207	0.188	−0.070	0.662	−0.263	0.093		
	**Non-PE**	0.142	0.311	0.094	0.504	0.252	0.069		
**PHOSPHATIDYLCHOLINES**									
PC (16:0/16:0)	**All**	−0.664	**<0.001**	−0.400	**<0.001**	−0.733	**<0.001**	NA	NA
	**PE**	−0.688	**<0.001**	−0.368	**0.016**	−0.713	**<0.001**		
	**Non**-**PE**	−0.232	0.094	0.137	0.327	−0.415	**0.002**		
PC (16:0/18:1)	**All**	−0.493	**<0.001**	−0.271	**0.008**	−0.584	**<0.001**	NA	NA
	**PE**	−0.54	**<0.001**	−0.258	0.099	−0.601	**<0.001**		
	**Non**-**PE**	0.025	0.854	0.035	0.806	−0.178	0.201		
PC (16:1/18:1)	**All**	−0.412	**<0.001**	−0.225	**0.028**	−0.490	**<0.001**	NA	NA
	**PE**	−0.347	**0.024**	−0.098	0.537	−0.441	**0.003**		
	**Non**-**PE**	−0.055	0.697	−0.078	0.581	−0.137	0.327		

also includes the current information available on maternal-fetal transfer and previous literature on the main metabolites detected in the study.

## Discussion

PE is a complex disorder affecting the health of both mother and newborn. To our knowledge, this study is the first one to apply LC-MS based metabolomics to determine whether the metabolic profile of plasma from umbilical cord differs between infants born to PE and non-PE pregnancies. Our data clearly demonstrate that this strategy provides a global picture of the widespread metabolic alterations associated with PE and provides grounds for future studies. The main identified changes in PE newborns compared with non-PE newborns included increased levels of carnitine precursors and trimethylated compounds. All main findings of identified metabolites are further discussed below. However, we are not able to demonstrate whether these or other observed changes are a consequence of PE or do they play a role in the pathogenic process itself. They may also represent maternal and/or fetal compensatory mechanisms e.g. due to oxidative stress.

### Carnitine and acylcarnitines

The observed increased levels of carnitine, precursors of carnitine and acylcarnitines indicate that the carnitine biosynthesis and mitochondrial β-oxidation processes are altered in the PE. Previously, abnormalities of lipid metabolism, particularly impaired fatty acid oxidation, are recognized features of PE and are thought to contribute through endothelial dysfunction leading to vascular remodeling and atherosclerosis^17^. Very recent findings provide further evidence that the compromised lipid metabolism in PE may result from dysfunctional mitochondria^8^. Previously it has been shown that plasma carnitine concentrations are significantly higher in preterm infants than in term ones and there is a significant negative correlation between gestational age and plasma carnitine concentration^18^. We also observed this association between carnitine levels and gestational weeks but only in PE infants.

Carnitine is responsible for the transport of lipids from the cytoplasm into the mitochondria for energy metabolism. It is made primarily in the liver and kidneys, which are both significantly affected in PE^19^. Carnitines inhibit oxidative stress and prevent lipid peroxidation. Both of these are also important pathological processes in PE. Moreover, adequate levels of carnitine are crucial in the neonatal period, since the mitochondrial oxidation of fatty acids is essential for meeting the tremendous energy needs of newborns who have very little stored glycogen^20^.

Previously, Ilsinger *et al*.^21^ have shown free carnitine to be higher in the newborns of PE women. They have also demonstrated that the concentration of carnitine was higher in the PE newborns than in the maternal samples. Carnitine supply is thought to be primarily met through placental carnitine uptake from the maternal circulation^22^. Alternatively, higher carnitine levels in infants from PE pregnancies may also result from upregulated fetal or placental biosynthesis^23^. To our knowledge this is a first study to report elevated levels of carnitine precursors (trimethyllysine and gamma-butyrobetaine) in the cord plasma of newborns of PE pregnancy. In addition to microbiota dependent metabolism from carnitine, gamma-butyrobetaine is also produced endogenously from trimethyllysine^24^. Whether the increased concentrations observed in PE newborns reflect the increased need of carnitine or some other mechanism, remain to be elucidated. Currently, the knowledge on the role of carnitine precursors in different diseases is sparse. However, elevated serum levels of both gamma-butyrobetaine and trimethyllysine are shown to be associated with increased risk of cardiovascular death^25^. Furthermore, very recently Strand *et al*.^26^ demonstrated that serum levels of gamma-butyrobetaine and trimethyllysine predict long-term risk of type 2 diabetes independently of traditional risk factors, possibly reflecting dysfunctional fatty acid metabolism.

In addition to the various carnitine metabolites, we also detected increased levels of other trimethylated compounds, namely choline, glycine betaine and 5-AVAB in the PE pregnancies when compared to the non-PE newborns. A recent investigation described a group of trimethylated compounds, including 5-AVAB, to be associated with consumption of diets rich in whole-grains^27^. Furthermore, 5-AVAB has been shown to inhibit carnitine transporter OCTN2 dependent intake of L-carnitine in mouse and human cultured cardiomyocytes^28,29^. OCTN2 is also responsible for carnitine intake in the placental tissue^22^. In general the clear increase in various trimethylated compounds may indicate the fetus’s high demand for choline and methyl donors, as has been suggested also earlier^30^. Previously, the rise in maternal plasma choline concentrations during pregnancy has been shown to reflect the mobilization of maternal hepatic choline stores and increase in estrogen. In rats and mouse models it has been shown that high choline intake during gestation and early postnatal development improves cognitive function in adulthood and prevents age-related memory decline^31^.

Carnitine has also a role in removing excess toxic metabolites, e.g. acyl groups which are excreted as acylcarnitines by the kidneys. We also observed elevated levels of acylcarnitines (buturylcarnitine and octenoylcarnitine). Acylcarnitines have been previously investigated in maternal samples as potential biomarkers for PE throughout the pregnancy^12^. Whereas to our knowledge only Illsinger *et al*.^21^ has shown that impaired mitochondrial function and reduced capacity of fatty acid oxidation leads to accumulation of acylcarnitines in the newborns.

### Urea cycle

The observed increased levels of urea, creatine, creatinine, homocitrulline and guanidinopropionate in the cord plasma samples of the PE newborns indicate altered function of the urea cycle compared to the non-PE newborns. Urea and creatinine levels are known for long to be increased in women with PE^32,33^. Roopnarinesingh *et al*.^32^ have also reported elevated levels of urea and creatinine in the amniotic fluid of PE women, despite an insignificant increase in urea and creatinine concentrations in maternal and fetal plasma. In line with our results, Sharma *et al*.^33^ have demonstrated increased urea concentrations in the umbilical cord blood of infants born to women with severe PE. Thus, it is possible that these changes in the cord blood reflect a derangement in the mechanism required for the elimination of these metabolites from the maternal and/or from the fetal compartment.

### Phosphatidylcholines (PCs)

PCs are glycerophospholipids, where phosphorylcholine moiety occupies a glycerol substitution site. They are major component of biological membranes. In previous studies PC content in placental tissue of PE-women has been observed to be higher than in placental tissue of non-PE women^34^. We detected PC(16:1/18:1), PC(16:0/16:0), and PC(16:0/18:1) to be increased in cord plasma of PE newborns. 16:0 most likely represent palmitic acid; 16:1 palmitoleic acid and 18:1 vaccenic acid, elaidic or oleic acid. This may be related to the inflammatory response present in PE, since it has been indicated that among acyl-lysophospholipid species, lysoPC16:0 and lysoPC18:0 exert the greatest pro-inflammatory activities, such as cytokine secretion^35^. On the other hand, PC(16:0/16:0) is known to have a central role in the function of pulmonary surfactant^36^.

Lindsay *et al*.^37^ have speculated earlier that during normal pregnancy increasing maternal plasma concentrations of PCs indicate the enhanced role of phospholipids in non-esterified fatty acids transport to the fetus as pregnancy progresses. Whereas Schott *et al*.^38^ have stated that PE is a disorder in phospholipid metabolism in which malfunctioning of cellular membranes seems to play a major pathogenic role. These alterations could be a source of placental pathological changes in PE, such as lipid peroxide insult or dysregulation of lipid transport across the syncytiotrophoblast^34^. A high negative correlations between PCs, birth weight and SD observed in this study would strengthen this view.

### 4-pyridoxic acid

4-pyridoxic acid is a vitamin B6 catabolite excreted into the urine^39^. Levels are reported to be elevated in renal insufficiency and have a strong, positive correlation with plasma urea and creatinine^40^. Clearances of both 4-pyridoxic acid and creatinine increase during normal pregnancy presumably resulting from increased renal perfusion. Thus, we could speculate that the observed decreased concentrations in PE newborns may be due impaired maternal renal perfusion. On the other hand, Kliger *et al*.^41^ have shown already several decades ago that PE (“toxemic”) placentas are characterized by marked pyridoxine deficiency due to lowered pyridoxal kinase activity in the placenta as compared with that observed in the normal organ.

### 2PY

N-methyl-2-pyridone-5-carboxamide (2PY) is a major metabolite of nicotinamide and it has been identified as a uremic toxin^42^. To our knowledge, this is first study to report this compound in pregnancy and in PE. Previously, it has been shown that the accumulation of 2PY occur in renal failure and might contribute directly to the signs and symptoms of uremia^43^. Thus, one could speculate that the elevated levels observed in the cord plasma of PE newborns may be related to the impaired maternal/fetal renal function. Interestingly, 2PY is also an inhibitor of the poly(ADP-ribose) polymerase, which is involved in regulation of DNA replication and repair^43^. However, its role in PE remains to be elucidated.

### Polyamines

We detected two putrescine metabolites, acetylputrescine and 4-acetamidobutanoate to be increased in the cord plasma of PE newborns. Putrescine belongs to the group of polyamines which are essential for early embryonic development and successful pregnancy outcome^44^. They exert effects that include stimulation of cell division and proliferation, gene expression for the survival of cells, DNA and protein synthesis, regulation of apoptosis, oxidative stress, angiogenesis, and cell-cell communication activity^44^. Their role in PE is very little studied. Nitric oxide (NO) and polyamines are both products of L-arginine metabolism and it is thought that the L-arginine-NO-polyamine pathway may have a physiological role during pregnancy. To our knowledge, only Sooranna *et al*.^45^ have measured polyamine concentrations in first trimester and term placentae from normal and PE pregnancies, but no difference was observed.

### Tryptophan metabolites

We observed levels of two tryptophan metabolites, indolelactic acid and indolecarboxylic acid to be elevated in the cord plasma of PE newborns. The essential amino acid tryptophan is particularly important in pregnancy, because of the increased demand for maternal protein synthesis and fetal requirements for growth and development^46^. Previous studies have shown that indoleamine 2,3-dioxygenase (IDO), an immunosuppressive enzyme that converts tryptophan to kynurenine, is expressed in the placenta and might play a role in the maintenance of pregnancy, although its associations with the pathogenesis of PE remain unclear^47^. Indolecarboxylic acid is a known inhibitor of lipid peroxidation and therefore increased levels observed in PE could be associated with the altered mitochondrial function also observed. Indolelactic acid is less studied but Morita *et al*.^48^ have shown indolelactic acid levels to be higher in umbilical plasma than in maternal plasma during normal pregnancy.

Very recently, Gomez-Arango *et al*.^49^ studied placental microbiome of pregnant overweight/obese women and found that pathways encoding tryptophan were highly enriched. Catabolism of tryptophan in the placenta is linked with the establishment and maintenance of the feto-maternal immune tolerance, placental circulation and growth and modulation of antimicrobial activity by inhibiting ascending infections from the vagina.

The results obtained from this study demonstrates the complexity of metabolic alterations and value of non-targeted metabolomic approach in evaluating the pathophysiology of PE. Although changes in single metabolites do not fully represent the underlying process, they provide a basis for further studies. Although it should be borne in mind that differences observed e.g. in carnitine metabolism represent multiple metabolites and thus systemic alterations could be expected. However, it remains unclear whether these changes are a consequence of PE or whether they play a key role in the pathogenic process itself. Furthermore, the changes observed may be due to small actual or relative birth weight of the PE newborns. Interestingly, very recently Robinson *et al*.^50^ observed in line with our results a negative association between birth weight, indolelactic acid and PCs in a population based study. The additional effect of PE could be controlled by comparing results with small for gestational age, non-PE newborns. However, within this cohort we did not have such control newborns available. One could also speculate that feto-placental blood volume condition such as fetal hypovolemic state might have affected the results. However, the policies for timing of cord clamping were similar and the umbilical cord blood hemoglobin concentrations were within reference value in both groups (data not shown). Thus, we consider that the volume distribution does not explain the observed differences in metabolites.

Although the study contains relatively good number of samples, it lacks a validation procedure. In future with greater sample size it would be of interest to stratify PE women e.g. by parity, onset of PE or by the delivery mode. Moreover, in future it would of interest to explore whether the observed metabolic changes persist into later life and reflect the susceptibility to metabolic diseases in later life.

Maternal smoking, an important confounding factor, was an exclusion criteria in the present study. We were not able to control for maternal diet, because we did not have sufficient dietary data available to assess the effect. Furthermore, betamethasone was used for some of the PE women for the enhancement of fetal lung maturation when preterm delivery was imminent. There is possibility that the betamethasone treatment of those PE women have affected the metabolome of these infants. Furthermore, one could speculate that six of the PE women who suffered also from gestational diabetes may affects the results. Thus, the analyses were also re-performed without these mothers but only minor changes were observed (analyses not shown).

In conclusion, we clearly demonstrate that the LC-MS based strategy provides a global picture of the metabolic alterations associated with the PE newborns. We particularly observed enhanced carnitine biosynthesis and increased concentration of methyl donors. Whether these metabolic changes during early postnatal life are a consequence of PE or play a role in the pathogenic process itself remain to be elucidated.

## Electronic supplementary material

Molecular features with p-value below 0.05 (ordered by p-value)
