# Supplementary material for: A Non-Targeted LC-MS Profiling Reveals Elevated Levels of Carnitine Precursors and Trimethylated Compounds in the Cord Plasma of Pre-Eclamptic Infants
Source: Sci Rep. 2018 Oct 2;8:14616. doi: 10.1038/s41598-018-32804-5 (PMC6168522; doi:10.1038/s41598-018-32804-5)
Supplement: Supplementary file 1 — Molecular features with p-value below 0.05 (ordered by p-value) [file 41598_2018_32804_MOESM1_ESM.pdf]

Supplementary information

A NON-TARGETED LC-MS PROFILING REVEALS ELEVATED LEVELS OF CARNITINE PRECURSORS AND TRIMETHYLATED COMPOUNDS IN THE CORD PLASMA OF PRE-ECLAMPTIC INFANTS

Tiina Jääskeläinen, Olli Kärkkäinen, Jenna Jokkala, Kaisa Litonius, Seppo Heinonen, Seppo Auriola, Marko Lehtonen, Kati Hanhineva, Hannele Laivuori for the FINNPEC

Supplementary table 1: Molecular features with p-value below 0.05 (ordered by p-value)

								Control		Preeclampsia		t-test	Cohen's	PLS-DA	fold
Molecular feature	HILIC/RP	Ionization mode	Mass	RT	Identification	ID level	MSMS fragmentation	Mean	SD	Mean	SD	p	d	VIP	change
246.0849@1.5598935	HILIC	-	246.0849	1.56				219516	31762	285503	50192	1.41E-11	1.61	2.84	1.30
99.0428@1.7425824	HILIC	+	99.0428	1.74				37273	15349	70305	35484	3.97E-11	1.30	2.58	1.86
189.1115@6.1605635	HILIC	+	189.1115	6.16	Homocitrulline	2	Hilic (+) 10ev: 190.1182 --> 173.0915 (100), 127.0868 (98), 84.0828 (46), 190.1242 (23)	21621	8722	50291	35414	1.09E-10	1.30	2.29	2.07
60.0322@0.96027136	HILIC	+	60.0322	0.96	Urea	1	Hilic (+) 10ev: 61.0393 --> 44.0126 (100), 61.0391 (63)	128725	27028	177308	36079	1.88E-10	1.54	2.82	1.38
309.0806@1.5604054	HILIC	-	309.0806	1.56				56028	15907	74561	11789	4.88E-10	1.34	2.58	1.25
161.0688@3.2028368	HILIC	-	161.0688	3.20	Indolecarboxylic acid	2	Hilic (-) 20ev: 160.0613 --> 74.0245 (100), 98.0249 (54)	49467	7949	64244	13873	1.07E-09	1.35	2.51	1.29
188.0451@3.3214035	HILIC	-	188.0451	3.32				154224	31668	198520	37724	9.71E-09	1.28	2.50	1.29
129.0538@2.0377033	HILIC	+	129.0538	2.04				25895	14683	49431	27734	1.74E-08	1.11	2.30	1.84
143.0945@4.1020107	HILIC	+	143.0945	4.10				140675	99995	306714	183385	3.49E-08	1.17	2.30	2.35
466.1327@1.8160769	HILIC	-	466.1327	1.82				112516	45244	60772	40799	5.21E-08	-1.20	2.26	-2.23
169.0851@6.2600594	HILIC	+	169.0851	6.26	1-methylhistidine	1	Hilic (+) 10ev: 170.0928 --> 124.0871 (100), 170.0926 (39), 109.0757 (8), 96.0679 (5), 83.0615 (3)	1012731	241061	1419330	462953	6.75E-08	1.16	2.26	1.38
152.0586@1.495875	RP	+	152.0586	1.50				50515	24223	86975	32820	6.84E-08	1.28	2.49	1.78
128.0583@5.6848617	HILIC	+	128.0583	5.68				222708	65352	357427	176562	7.01E-08	1.11	2.06	1.52
123.0794@6.259089	HILIC	+	123.0794	6.26	Fragment of 1-methylhistidine			74650	17929	105873	36551	1.12E-07	1.15	2.26	1.39
152.0584@0.757505	HILIC	+	152.0584	0.76	Tentative 2PY (N-methyl-2-pyridone-5-carboxamide)	3	Hilic (+) 20ev: 153.0661 --> 108.0451 (100), 153.0661 (95), 92.0501 (95), 136.0392 (93), 110.0589 (63), 53.0389 (42), 39.0227 (17), 80.0486 (16)	248111	115913	412210	144650	1.26E-07	1.26	2.47	1.72
135.0319@0.7583886	HILIC	+	135.0319	0.76	Fragment of tentative 2PY			262332	124448	437700	154689	1.42E-07	1.26	2.47	1.73
72.0575@0.73860157	HILIC	-	72.0575	0.74	Hydroxyisovaleric acid [M-COOH]	2	Hilic (-) 10ev: 117.0557 --> 71.0495 (100), 117.0569 (65), 80.0324 (12)	240253	65210	325401	101084	1.51E-07	1.02	2.03	1.39
258.0852@1.1630198	HILIC	-	258.0852	1.16				49302	9278	63488	14029	1.55E-07	1.22	2.37	1.28
182.058@1.0825481	HILIC	-	182.0580	1.08	p-Hydroxyphenyllactic acid	2	Hilic (-) 20ev: 181.0508 --> 135.0453 (100), 57.9755 (71), 119.0506 (47)	55698	21852	82086	30516	5.16E-07	1.01	2.04	1.48
113.0588@1.2412335	HILIC	+	113.0588	1.24	Creatinine	1	Hilic (+) 10ev: 114.0664 --> 114.0667 (100), 44.0495 (79), 86.0714 (11)	14061144	2744481	17035158	2972637	1.33E-06	1.04	2.14	1.22
563.4307@1.1281083	HILIC	+	563.4307	1.13				78369	22577	60826	14791	1.56E-06	-0.94	1.84	-1.31
512.2974@3.2875962	HILIC	-	512.2974	3.29				207976	78238	337772	158474	1.84E-06	1.10	2.33	1.56
500.2078@5.987612	RP	-	500.2078	5.99				108305	36145	69529	40412	2.43E-06	-1.01	2.02	-1.72
286.2371@5.938252	HILIC	+	286.2371	5.94	Diacetylspermidine	2	Hilic (+) 10ev: 287.2440 --> 287.2441 (100), 171.1489 (98), 100.0756 (51), 269.2333 (13), 112.1123 (4)	192954	81290	374328	240967	2.63E-06	1.13	2.06	1.75
366.1498@7.378337	RP	-	366.1498	7.38				66635	22422	44197	18537	3.02E-06	-1.10	2.12	-1.57
822.5734@12.990248	RP	-	822.5734	12.99				219576	38344	267478	50409	3.82E-06	1.08	2.12	1.22
145.1101@3.3068821	HILIC	+	145.1101	3.31	gamma-butyrobetaine	1	Hilic (+) 10ev: 146.1173 --> 146.117 (100), 87.0442 (47), 60.0809 (41), 43.0175 (8), 70.0649 (6)	350527	87343	493931	208361	4.39E-06	0.97	1.89	1.36
182.0579@2.7312887	RP	-	182.0579	2.73				70174	28922	96540	30247	4.84E-06	0.89	1.93	1.41
131.0585@1.5718465	HILIC	-	131.0585	1.57	b-Guanidinopropionate	2	Hilic (-) 10ev: 130.0511 --> 88.0409 (100), 130.0503 (31), 116.0212 (28)	57015	10798	68237	13212	6.11E-06	0.93	1.99	1.20
260.0986@5.747193	HILIC	-	260.0986	5.75				57267	11797	69977	14310	6.99E-06	0.97	2.13	1.22
524.076@2.3701942	HILIC	+	524.0760	2.37				29550	13254	17192	11685	7.09E-06	-0.99	1.98	-1.70
205.0738@0.71782726	HILIC	-	205.0738	0.72	Indolelactic acid	2	Hilic (-) 10ev: 204.0666 --> 204.0666 (100), 158.0636 (53), 116.0524 (43), 58.1373 (19), 186.0521 (19)	80418	21392	104621	25444	7.83E-06	1.03	2.27	1.31
991.5735@12.913568	RP	-	991.5735	12.91				558044	45833	508688	55262	8.44E-06	-0.98	2.07	-1.10
158.0437@1.8829517	HILIC	-	158.0437	1.88				122831	29254	160583	50210	8.67E-06	0.95	1.95	1.29
183.0528@0.84989387	HILIC	-	183.0528	0.85	4-pyridoxic acid	2	Hilic (-) 10ev: 182.0455 --> 138.0569 (100), 182.0453 (80), 108.0451 (50), 108.3154 (15)	147825	118474	73118	52861	1.01E-05	-0.87	1.70	-2.00
205.074@4.5351343	RP	+	205.0740	4.54				147899	41180	190284	44158	1.10E-05	0.99	2.22	1.31
506.2335@8.663646	RP	-	506.2335	8.66				433042	358622	262981	136275	1.12E-05	-0.69	1.34	-1.64
1561.1267@12.334761	RP	-	1561.1267	12.33				355004	74607	483877	165678	1.35E-05	1.07	2.13	1.32
321.0804@1.1603175	HILIC	-	321.0804	1.16				18406	3783	23084	5521	1.38E-05	1.01	2.11	1.24
145.0741@0.767923	HILIC	-	145.0741	0.77	4-acetamidobutanoate	2	Hilic (-) 10ev: 144.0670 --> 100.0755 (100), 144.0658 (59), 102.0563 (38)	27105	7570	38724	18256	1.53E-05	0.90	1.87	1.36
803.568@12.334366	RP	-	803.5680	12.33	PC(18:1/16:1) [M+FA], PC 34:2	2	RP (-) 40ev: 281.2469 (100), 253.2193 (82)	11662142	1256335	13468494	2313587	1.62E-05	1.01	2.07	1.14
187.0457@5.747492	HILIC	-	187.0457	5.75				67216	9474	76504	10164	1.85E-05	0.95	2.14	1.14
119.0583@5.746241	HILIC	-	119.0583	5.75	Threonine	1	Hilic (-) 10ev: 118.0512 --> 74.0255 (100), 118.053 (16)	276105	77703	366950	120604	2.00E-05	0.92	1.98	1.31
565.4467@1.1069216	HILIC	+	565.4467	1.11				69288	18311	57155	12274	2.02E-05	-0.79	1.61	-1.23
173.999@2.3016925	RP	-	173.9990	2.30				202365	139143	419391	395306	2.08E-05	0.81	1.72	1.88
336.116@1.5590904	HILIC	-	336.1160	1.56				17208	7513	25022	7495	2.11E-05	1.04	2.11	1.32
480.1123@2.374615	HILIC	-	480.1123	2.37				90144	49722	52124	37469	2.20E-05	-0.87	1.76	-1.88
899.5867@12.989594	RP	-	899.5867	12.99				1316155	199004	1558888	290251	2.92E-05	0.99	2.02	1.18
1515.1259@12.341703	RP	+	1515.1259	12.34				1732514	427736	2487313	1034485	3.29E-05	1.03	2.11	1.36
745.5621@12.750534	RP	-	745.5621	12.75				84923	10790	99413	19494	3.49E-05	0.96	2.08	1.16
318.0015@3.4889998	HILIC	-	318.0015	3.49				24290	4442	28560	4853	3.55E-05	0.92	1.97	1.18
550.2539@3.3225248	HILIC	+	550.2539	3.32	Unknown adduct of gammabutyrobetaine			19125	9158	30476	15122	3.64E-05	0.93	2.10	1.55
130.1104@3.3570101	HILIC	+	130.1104	3.36	Acetylputrescine	1	Hilic (+) 10ev: 131.1176 --> 114.091 (100), 72.0803 (54), 131.1162 (26)	86232	28728	117788	48053	3.64E-05	0.82	1.81	1.34

								Control		Preeclampsia		t-test	Cohen's	PLS-DA	fold
Molecular feature	HILIC/RP	Ionization mode	Mass	RT	Identification	ID level	MSMS fragmentation	Mean	SD	Mean	SD	p	d	VIP	change
266.1549@0.953394	HILIC	-	266.1549	0.95				49681	13309	63979	17188	3.79E-05	0.94	1.95	1.29
831.6003@12.987326	RP	-	831.6003	12.99				5428486	899547	6532175	1358939	4.00E-05	0.98	2.02	1.19
743.5449@12.33603	RP	-	743.5449	12.34				82696	9642	95864	18158	4.13E-05	0.95	1.99	1.15
177.0457@1.2458937	HILIC	-	177.0457	1.25				25187	5372	29567	7112	4.14E-05	0.70	1.57	1.21
464.1175@1.1112592	HILIC	-	464.1175	1.11				311529	139380	195088	113375	4.48E-05	-0.92	1.84	-1.75
161.1052@4.852845	HILIC	+	161.1052	4.85	L-Carnitine	1	Hilic (+) 10ev: 162.1119 --> 162.1123 (100), 103.0387 (14), 60.0802 (12), 85.0278 (3)	8122873	2170320	11045709	4489046	4.73E-05	0.88	1.77	1.32
769.5469@12.642329	RP	-	769.5469	12.64				42353	29354	61464	36544	4.97E-05	0.58	1.48	1.27
386.1751@5.5915656	RP	-	386.1751	5.59				177012	70630	123302	98728	5.80E-05	-0.63	1.35	-1.69
178.0297@1.479471	HILIC	-	178.0297	1.48				56392	10294	67333	14214	5.94E-05	0.89	1.98	1.19
402.1702@0.88816506	HILIC	-	402.1702	0.89				102504	42904	67638	36395	6.90E-05	-0.88	1.76	-1.57
496.3022@2.134485	HILIC	-	496.3022	2.13				47198	27026	70176	28374	7.03E-05	0.83	2.01	1.53
75.0319@5.7464905	HILIC	-	75.0319	5.75	fragment of Threonine			36500	13618	50041	17291	7.04E-05	0.88	1.92	1.30
101.0835@4.8530583	HILIC	+	101.0835	4.85	fragment of L-Carnitine			21368	4936	28149	10843	7.75E-05	0.86	1.73	1.27
743.5851@13.106481	RP	+	743.5851	13.11	Plasmenyl-PC(34:1); PC(P-34:1)	2	RP(+) 10ev: 744.5911 --> 744.5892 (100), 184.0728 (15)	413034	153718	591165	230521	7.95E-05	0.82	1.75	1.42
281.1122@4.401932	HILIC	+	281.1122	4.40	1-methyladenosine	1	Hilic (+) 10ev: 282.1199 --> 150.0774 (100), 282.1203 (35), 45.4672 (4)	76609	14700	92584	20665	8.80E-05	0.90	1.87	1.20
591.4629@1.0897286	HILIC	+	591.4629	1.09				108512	28401	85884	21554	9.08E-05	-0.91	1.79	-1.26
829.584@12.52754	RP	-	829.5840	12.53				12074220	1754200	13680395	1970688	9.30E-05	0.86	1.83	1.13
225.075@1.6694564	HILIC	+	225.0750	1.67				57665	23247	75012	22827	0.00010	0.75	1.73	1.34
785.5937@13.000917	RP	+	785.5937	13.00	PC(36:2)	2	RP(+) 10ev: 786.6 --> 786.6012 (100), 184.0743 (15)	13926389	3090343	17356082	4344228	0.00010	0.92	1.98	1.24
102.0319@4.8538547	HILIC	+	102.0319	4.85	fragment of L-Carnitine			38454	9997	50865	19856	0.00011	0.83	1.71	1.29
450.1381@1.3135197	HILIC	-	450.1381	1.31				659764	171221	505954	239646	0.00011	-0.75	1.58	-1.43
231.1467@1.5653496	HILIC	+	231.1467	1.57	Butyrylcarnitine	1	Hilic (+) 10ev: 232.1542 --> 232.1542 (100), 85.0283 (63), 173.0814 (21), 60.0789 (16), 144.1018 (7)	288894	117728	459566	303568	0.00012	0.81	1.72	1.48
384.1606@6.0700865	RP	-	384.1606	6.07				169114	76119	109916	62081	0.00012	-0.86	1.72	-1.65
195.1104@1.4662216	HILIC	-	195.1104	1.47				84519	22385	121685	72651	0.00012	0.78	1.52	1.35
432.216@0.9517113	HILIC	-	432.2160	0.95				128220	41278	166383	47393	0.00013	0.86	2.08	1.33
757.5629@12.343165	RP	+	757.5629	12.34	PC(18:1/16:1), PC(34:2)	2	RP(+) 10ev: 758.5704 --> 758.5688 (100), 184.0735 (15)	38555056	5331233	44895722	9017731	0.00013	0.88	1.95	1.15
285.1941@4.9741244	RP	+	285.1941	4.97	Octenoylcarnitine	3	20ev: 286.2014 --> 85.0285 (100), 60.0807 (9), 144.1012 (7), 57.0336 (6)	97790	47363	139581	68457	0.00013	0.72	1.56	1.43
510.1592@3.2422845	HILIC	-	510.1592	3.24				78675	51151	47746	32465	0.00014	-0.74	1.52	-1.76
873.5713@12.740996	RP	-	873.5713	12.74				2085018	221514	2301600	287135	0.00015	0.85	1.91	1.10
285.1943@0.94856346	HILIC	+	285.1943	0.95				277063	132217	381663	167103	0.00015	0.70	1.49	1.38
416.3293@10.149903	RP	+	416.3293	10.15				75003	36940	138778	102970	0.00016	0.91	1.75	1.62
805.5842@12.755218	RP	-	805.5842	12.76	PC(16:0/18:1) [M+FA], PC 34:1	2	RP (-) 20ev: 804.577 --> 744.555 (100), 804.5764 (30), 281.2487 (27), 255.2333 (5)	11370762	1470663	13025363	2348092	0.00016	0.87	1.97	1.14
779.5672@12.644232	RP	-	779.5672	12.64	PC(16:0/16:0) [M+FA], PC 32:0	2	RP (-) 20ev: 778.5606 --> 718.5397 (100), 778.5605 (40), 255.2282 (17)	471630	116628	651965	257154	0.00017	0.96	2.09	1.31
1565.1606@12.752208	RP	-	1565.1606	12.75				267392	67881	355092	127825	0.00019	0.90	2.00	1.29
203.1157@3.121567	HILIC	+	203.1157	3.12				29401	17957	41628	24765	0.00022	0.57	1.30	1.58
85.0528@0.77546597	HILIC	+	85.0528	0.78				22610	6407	28816	9918	0.00022	0.76	1.67	1.26
89.0475@5.5390854	HILIC	-	89.0475	5.54				26025	6485	34524	13996	0.00022	0.83	1.71	1.27
717.5315@11.999971	RP	+	717.5315	12.00				85813	58913	125206	85961	0.00023	0.54	1.53	1.34
119.0584@5.765933	HILIC	+	119.0584	5.77	Aminohydroxybutyric acid	2	Hilic (+) 10ev: 120.0655 --> 74.0599 (100), 56.0493 (86), 102.0543 (32), 120.065(13), 84.0446 (11)	922498	266109	1185177	361973	0.00023	0.84	1.85	1.28
430.2364@9.68475	RP	-	430.2364	9.68				77417	25876	52650	25835	0.00024	-0.96	2.08	-1.34
284.1203@5.53872	HILIC	-	284.1203	5.54				23348	9255	34946	19317	0.00024	0.81	1.70	1.42
206.1991@1.4785776	HILIC	+	206.1991	1.48				29931	17646	80923	145931	0.00026	0.62	1.12	1.80
228.1472@4.1124287	HILIC	+	228.1472	4.11				136160	85344	267703	323707	0.00027	0.64	1.31	1.57
259.9859@5.7485585	HILIC	-	259.9859	5.75				30256	7460	36914	9295	0.00029	0.79	1.80	1.22
288.0395@1.235573	HILIC	+	288.0395	1.24	Unknown adduct of creatinine			130993	13874	141623	12704	0.00030	0.80	1.81	1.08
537.3792@1.1531796	HILIC	+	537.3792	1.15				61354	31423	41475	28977	0.00030	-0.66	1.39	-1.45
959.4498@1.1257075	HILIC	-	959.4498	1.13				102781	40732	65837	40049	0.00031	-0.91	1.89	-1.53
967.5719@12.994427	RP	-	967.5719	12.99				371398	120717	458736	153325	0.00031	0.64	1.37	1.21
783.5791@12.538605	RP	+	783.5791	12.54				44239006	8530708	51643467	10156547	0.00031	0.79	1.79	1.17
90.0319@1.4661254	HILIC	-	90.0319	1.47				459420	111589	593612	250776	0.00033	0.74	1.53	1.25
153.051@5.5389123	HILIC	-	153.0510	5.54				74532	11622	85238	15612	0.00035	0.79	1.69	1.14
131.0695@5.560988	HILIC	+	131.0695	5.56	Creatine	1	Hilic (+) 10ev: 132.0715 --> 90.0546 (100), 132.0765 (81), 44.0491 (44), 86.0596 (17), 132.0665 (9)	3855742	849800	4760167	1463396	0.00037	0.78	1.66	1.21
166.0629@0.70108867	HILIC	-	166.0629	0.70				22761	15155	35410	18830	0.00037	0.74	1.60	1.32
166.0476@6.103942	HILIC	-	166.0476	6.10				43333	17041	56136	22291	0.00038	0.65	1.54	1.36
795.5566@12.741263	RP	-	795.5566	12.74				864839	81791	938472	105661	0.00040	0.79	1.89	1.08
239.1367@1.4664137	HILIC	-	239.1367	1.47				115504	34498	161191	93990	0.00041	0.71	1.43	1.33
983.45@1.1137738	HILIC	-	983.4500	1.11				84717	36244	54138	31302	0.00042	-0.91	1.79	-1.38
450.1379@1.0361383	HILIC	-	450.1379	1.04				27358	13526	17153	13005	0.00042	-0.77	1.61	-1.52
177.0166@5.747683	HILIC	-	177.0166	5.75				52805	6679	58609	8522	0.00044	0.76	1.80	1.11
264.9736@4.404616	RP	+	264.9736	4.40				65073	17032	52692	14127	0.00044	-0.79	1.63	-1.23

								Control		Preeclampsia		t-test	Cohen's	PLS-DA	fold
Molecular feature	HILIC/RP	Ionization mode	Mass	RT	Identification	ID level	MSMS fragmentation	Mean	SD	Mean	SD	p	d	VIP	change
131.0693@5.5388265	HILIC	-	131.0693	5.54	Creatine	1	Hilic (-) 10ev: 130.0618 --> 88.0398 (100)	39374	12476	54509	27341	0.00045	0.76	1.63	1.32
188.1522@6.758182	HILIC	+	188.1522	6.76	Trimethyllysine	1	Hilic (+) 10ev: 189.1595 --> 189.1582 (100), 130.0877 (42), 84.0809 (40), 60.0808 (23)	117516	38938	146709	44830	0.00045	0.70	1.60	1.25
759.578@12.764939	RP	+	759.5780	12.76	PC(16:0/18:1), PC(34:1)	2	RP(+) 10ev: 760.5855 --> 760.5833 (100), 184.0734 (15)	33449490	5259530	39161086	8790871	0.00045	0.81	1.91	1.16
555.3528@10.409204	RP	-	555.3528	10.41				113884	38154	92555	32174	0.00047	-0.61	1.30	-1.25
701.3104@10.11647	RP	-	701.3104	10.12				49859	7694	55603	6930	0.00047	0.79	1.73	1.12
743.5463@12.103155	RP	+	743.5463	12.10				344252	69973	456136	211227	0.00047	0.80	1.78	1.25
97.9676@7.7432446	HILIC	-	97.9676	7.74	Sulfuric acid	2	Hilic (-) 20ev: 96.9600 --> 96.9600 (100), 79.9579 (82)	925437	140826	1041549	178657	0.00048	0.73	1.51	1.12
869.5707@12.7919	RP	-	869.5707	12.79				231209	62796	170921	80401	0.00049	-0.84	1.77	-1.30
159.1258@2.0653882	HILIC	+	159.1258	2.07	5-AVAB	1	Hilic (+) 10ev: 160.1335 --> 160.1333 (100), 101.0602 (18), 60.0807 (14), 55.0536 (3)	669078	236375	956325	508650	0.00049	0.77	1.60	1.43
103.0999@1.4785815	HILIC	+	103.0999	1.48	Choline	2	Hilic (+) 10ev: 104.1071 --> 104.1071 (100), 60.0809 (21), 45.0336 (6), 58.0653 (2)	29777821	6104113	36291027	10781073	0.00051	0.77	1.62	1.20
432.3241@9.673707	RP	-	432.3241	9.67				255309	73637	197170	68253	0.00052	-0.82	1.69	-1.33
368.1664@7.405428	RP	-	368.1664	7.41	Unknown steroid	3	RP (-) 20ev: 367.1602 (100), 96.9592 (45)	2622852	883580	1943962	923171	0.00057	-0.75	1.60	-1.48
257.1627@1.1977626	HILIC	+	257.1627	1.20				20878	13622	30609	15489	0.00058	0.67	1.48	1.49
261.1576@3.4491658	HILIC	+	261.1576	3.45				29082	11003	40828	21223	0.00058	0.73	1.54	1.35
733.562@12.655955	RP	+	733.5620	12.66	PC(16:0/16:0), PC(32:0)	2	RP(+) 10ev: 734.5709 --> 734.5717 (100), 184.074 (15)	1243080	387675	1808758	841770	0.00058	0.92	2.05	1.37
101.0474@5.7659316	HILIC	+	101.0474	5.77				98304	30877	124446	42545	0.00058	0.71	1.59	1.26
193.1309@1.4671832	HILIC	-	193.1309	1.47				200728	56357	265500	123697	0.00059	0.72	1.49	1.28
468.2366@1.3524104	HILIC	-	468.2366	1.35				39594	18914	29456	13750	0.00060	-0.62	1.35	-1.40
414.3134@9.678997	RP	+	414.3134	9.68				135821	40137	104481	37706	0.00060	-0.81	1.67	-1.34
907.5898@12.801803	RP	-	907.5898	12.80				137356	49205	164387	61371	0.00060	0.49	1.09	1.19
464.1167@1.342742	HILIC	-	464.1167	1.34				44401	19978	29437	19467	0.00065	-0.76	1.63	-1.46
635.3408@1.2119424	HILIC	-	635.3408	1.21				55828	6595	60802	7442	0.00067	0.71	1.56	1.09
957.5443@12.997534	RP	-	957.5443	13.00				131812	34346	158154	38730	0.00069	0.72	1.56	1.20
1068.6176@10.100798	RP	-	1068.6176	10.10				132273	33822	108357	30856	0.00070	-0.74	1.61	-1.23
877.5756@13.618411	RP	+	877.5756	13.62				59886	37578	94300	38892	0.00071	0.90	1.85	1.38
731.5472@12.202503	RP	+	731.5472	12.20	PC(32:1)	2	RP(+) 10ev: 732.555 ---> 732.5525 (100), 184.073 (15)	4932763	1336518	6678008	2869699	0.00074	0.83	1.89	1.29
285.0963@1.3852043	HILIC	+	285.0963	1.39				24734	10099	30096	8838	0.00076	0.57	1.32	1.25
202.1317@4.681564	HILIC	+	202.1317	4.68				146704	63072	211812	126258	0.00077	0.69	1.54	1.36
947.5854@12.792761	RP	-	947.5854	12.79				695238	183043	566766	193091	0.00078	-0.68	1.67	-1.26
823.5415@13.000701	RP	+	823.5415	13.00				179790	80682	225027	88020	0.00080	0.54	1.25	1.27
710.5132@11.6102085	RP	-	710.5132	11.61				74296	11026	84358	15397	0.00085	0.76	1.70	1.13
402.1711@6.1199713	RP	-	402.1711	6.12				123339	49374	92386	46773	0.00089	-0.64	1.35	-1.44
275.1734@2.3287966	HILIC	+	275.1734	2.33				34498	24264	50193	31191	0.00089	0.57	1.33	1.46
899.5608@12.9377	RP	+	899.5608	12.94				427634	69367	408155	41631	0.00091	-0.35	0.89	-1.07
532.2649@4.1423697	HILIC	+	532.2649	4.14				34440	17911	49650	22452	0.00093	0.75	1.84	1.45
763.5478@0.9037337	HILIC	-	763.5478	0.90				390516	108690	443302	125987	0.00100	0.45	1.11	1.12
140.0586@5.288093	HILIC	+	140.0586	5.29				26395	22627	52022	47256	0.00111	0.73	1.69	1.71
141.0791@1.3203065	HILIC	+	141.0791	1.32				39427	43485	71462	66137	0.00113	0.58	1.42	1.84
297.1073@1.9449425	HILIC	+	297.1073	1.94				55838	12804	68021	19692	0.00113	0.75	1.65	1.20
751.5377@12.092802	RP	-	751.5377	12.09	PC(14:0/16:0) [M+FA], PC30:0	2	RP (-) 20ev: 750.5303 --> 690.5098 (52), 227.2052 (22), 255.2324 (20)	283870	60928	365203	134372	0.00115	0.83	2.01	1.23
582.3289@1.218067	HILIC	-	582.3289	1.22				162469	16843	175694	18448	0.00117	0.75	1.60	1.08
174.1367@6.6708083	HILIC	+	174.1367	6.67				45091	20320	63953	33816	0.00118	0.70	1.50	1.41
614.4884@13.653746	RP	+	614.4884	13.65				126784	56717	177222	97825	0.00120	0.65	1.44	1.36
494.1649@1.5609603	HILIC	-	494.1649	1.56				357057	197552	239130	139436	0.00124	-0.70	1.48	-1.61
508.2674@4.252737	HILIC	+	508.2674	4.25				19443	13174	35267	23536	0.00125	0.86	1.78	1.54
203.1158@2.9447	HILIC	+	203.1158	2.94	Acetylcarnitine	2	Hilic (+) 10ev: 204.1229 --> 204.1232 (100), 85.0285 (74), 145.05 (24), 60.0806 (18)	7074347	2864503	10152900	6004609	0.00128	0.69	1.53	1.36
796.5752@12.361108	RP	+	796.5752	12.36				100175	35541	74488	41833	0.00130	-0.66	1.62	-1.19
59.0733@1.4788655	HILIC	+	59.0733	1.48				31581	13026	42400	16600	0.00130	0.73	1.61	1.23
717.5664@12.956753	RP	+	717.5664	12.96				112513	28242	151615	66875	0.00132	0.82	1.86	1.27
803.5343@12.292024	RP	+	803.5343	12.29				3937894	576612	3556765	625699	0.00133	-0.63	1.53	-1.11
788.531@11.610996	RP	-	788.5310	11.61				141351	20771	161463	33370	0.00135	0.74	1.65	1.13
720.5418@11.609544	RP	-	720.5418	11.61				525374	76535	595918	116177	0.00137	0.73	1.64	1.12
144.0422@2.943805	HILIC	+	144.0422	2.94				29347	11054	40866	22203	0.00141	0.69	1.53	1.33
145.0737@0.7920776	HILIC	+	145.0737	0.79				97743	38684	149128	101057	0.00142	0.74	1.64	1.38
181.05@3.5151331	HILIC	+	181.0500	3.52				91614	78915	147705	107770	0.00146	0.60	1.30	1.87
845.5394@12.216898	RP	-	845.5394	12.22				198564	74454	263009	106474	0.00150	0.71	1.57	1.32
1583.1132@12.185694	RP	-	1583.1132	12.19				131864	29738	163631	54984	0.00151	0.75	1.75	1.21
255.0331@5.7502604	HILIC	-	255.0331	5.75				22426	3088	24414	2910	0.00156	0.66	1.63	1.09
150.1363@1.4995939	HILIC	+	150.1363	1.50				121244	210079	348711	985376	0.00160	0.38	0.88	2.12
777.5527@12.18503	RP	-	777.5527	12.19				1688483	448144	2265955	966542	0.00160	0.82	1.83	1.27
1549.1407@12.517332	RP	+	1549.1407	12.52				149531	44575	189436	77659	0.00166	0.65	1.57	1.27
705.5313@12.10256	RP	+	705.5313	12.10	PC(30:0)	2	RP(+) 10ev: 706.539 --> 706.5373 (100), 184.0732 (20)	729437	195475	995341	449697	0.00167	0.82	2.01	1.28
283.1628@1.4662933	HILIC	-	283.1628	1.47				209958	109341	344592	247834	0.00170	0.75	1.53	1.39

								Control		Preeclampsia		t-test	Cohen's	PLS-DA	fold
Molecular feature	HILIC/RP	Ionization mode	Mass	RT	Identification	ID level	MSMS fragmentation	Mean	SD	Mean	SD	p	d	VIP	change
169.0851@6.8600483	HILIC	+	169.0851	6.86				406033	384116	678822	573330	0.00178	0.57	1.42	1.84
993.4709@1.1858466	HILIC	-	993.4709	1.19				63640	36604	37223	31380	0.00183	-0.78	1.63	-1.47
811.4833@12.344175	RP	+	811.4833	12.34				74289	10047	83282	14402	0.00188	0.74	1.61	1.11
674.5364@11.624317	RP	+	674.5364	11.62	SM(32:1)	2	RP(+) 10ev: 675.5443 --> 675.5428 (100), 184.0736 (68)	865499	148563	999767	229291	0.00188	0.71	1.64	1.14
259.1787@1.0541074	HILIC	+	259.1787	1.05	Hexanoylcarnitine	1	Hilic (+) 10ev: 260.1853 --> 85.0285 (100), 260.1853 (91), 201.1126 (16), 60.0806 (13), 99.0784 (7)	159899	75167	243850	153408	0.00189	0.73	1.59	1.42
512.3094@0.9817533	HILIC	-	512.3094	0.98				29602	19999	48764	42192	0.00191	0.62	1.31	1.54
926.5379@1.1207309	HILIC	-	926.5379	1.12				50019	16342	60133	15504	0.00196	0.64	1.45	1.22
416.2241@7.8087955	RP	-	416.2241	7.81				1936291	1039391	2857927	1635604	0.00200	0.69	1.51	1.47
510.283@4.1090207	HILIC	-	510.2830	4.11				93291	37347	118231	41174	0.00200	0.64	1.72	1.29
913.5572@12.910612	RP	-	913.5572	12.91				305631	56756	285677	41029	0.00203	-0.41	0.97	-1.09
364.0982@6.392563	HILIC	+	364.0982	6.39				30829	16512	46419	34356	0.00206	0.61	1.33	1.40
132.0534@6.192008	HILIC	-	132.0534	6.19	L-Asparagine	1	Hilic (-) 20ev: 131.0460 --> 41.9980 (100), 70.0284 (53)	68316	14409	82134	24962	0.00208	0.70	1.47	1.18
185.0815@1.4724611	HILIC	-	185.0815	1.47				21206	3757	24373	5636	0.00212	0.67	1.44	1.14
204.0352@5.747501	HILIC	-	204.0352	5.75				42349	6707	47416	8416	0.00218	0.67	1.70	1.12
791.6037@13.284373	RP	-	791.6037	13.28				182782	45270	227568	67324	0.00219	0.80	1.78	1.19
150.0529@3.6811545	HILIC	-	150.0529	3.68				112319	39454	143339	61683	0.00235	0.61	1.48	1.26
1015.5713@12.805312	RP	-	1015.5713	12.81				143473	48227	118866	50783	0.00236	-0.50	1.62	-1.23
454.3058@9.679979	RP	+	454.3058	9.68				90898	27332	70144	27909	0.00238	-0.75	1.62	-1.30
825.5536@11.985963	RP	-	825.5536	11.99	PC(16:1/20:4) [M+FA], PC 36:5	2	RP (-) 20ev: 824.5458 --> 764.5202 (100), 303.2323 (27), 824.5466 (20),	2944841	1037713	3802877	1540620	0.00244	0.67	1.41	1.27
384.1604@6.2549796	RP	-	384.1604	6.25				9499091	3259910	7421983	3297859	0.00247	-0.63	1.45	-1.37
555.3109@1.2284697	HILIC	-	555.3109	1.23				166495	47022	187525	38778	0.00249	0.49	1.14	1.09
402.1692@0.90215373	HILIC	-	402.1692	0.90				94370	50001	65742	38200	0.00250	-0.65	1.38	-1.48
688.5511@11.826177	RP	+	688.5511	11.83	SM(33:1)	2	RP(+) 10ev: 689.5598 --> 689.5599 (100), 184.0732 (40)	275927	54248	322285	81432	0.00257	0.68	1.69	1.15
242.1263@1.8007017	HILIC	-	242.1263	1.80				61564	38404	81473	42645	0.00259	0.49	1.16	1.41
226.2298@10.429677	RP	-	226.2298	10.43				174130	57828	219821	65247	0.00266	0.74	1.59	1.17
384.161@6.4852567	RP	-	384.1610	6.49				238386	95136	179578	99667	0.00267	-0.60	1.30	-1.39
450.1385@1.1977354	HILIC	-	450.1385	1.20				147701	73005	103079	64369	0.00267	-0.65	1.38	-1.52
970.5419@13.021991	RP	+	970.5419	13.02				127448	33941	145474	47940	0.00275	0.44	1.16	1.14
436.1527@7.4064493	RP	-	436.1527	7.41				75864	21622	59853	24636	0.00275	-0.69	1.53	-1.29
609.5327@12.53233	RP	-	609.5327	12.53				204769	63913	289091	153660	0.00286	0.78	1.80	1.29
270.1982@7.3069563	RP	+	270.1982	7.31				74533	39383	59198	33428	0.00286	-0.42	1.01	-1.40
923.5873@12.894838	RP	-	923.5873	12.89				2212393	229263	2057130	258454	0.00296	-0.64	1.45	-1.08
272.2353@10.429668	RP	-	272.2353	10.43				166743	36987	198144	59840	0.00302	0.65	1.45	1.17
557.2252@12.561329	RP	-	557.2252	12.56				320049	60719	284748	54176	0.00302	-0.61	1.35	-1.12
316.2403@8.829803	RP	+	316.2403	8.83				184345	115986	266431	211407	0.00306	0.50	1.24	1.43
723.52@12.5317335	RP	-	723.5200	12.53	Plasmenyl-PE(36:4); PE(P-16:0/20:4)	2	RP(-) 20ev: 722.514 (100), 303.2325 (80), 436.2998 (6), 646.0751 (6)	193620	61664	274911	149734	0.00313	0.77	1.78	1.29
1198.7664@11.9912405	RP	+	1198.7664	11.99				1062403	419689	804949	290879	0.00314	-0.72	1.46	-1.32
891.5324@12.74091	RP	-	891.5324	12.74				103170	12236	112019	14537	0.00315	0.66	1.56	1.08
1513.112@12.248851	RP	+	1513.1120	12.25				142906	35391	185982	79224	0.00319	0.75	1.85	1.24
457.1689@10.656244	RP	-	457.1689	10.66				145251	44779	131207	43278	0.00320	-0.32	0.83	-1.18
595.363@1.0939316	HILIC	+	595.3630	1.09				68630	17306	56494	17930	0.00320	-0.69	1.63	-1.19
613.3387@10.081936	RP	-	613.3387	10.08	LysoPC(22:6) [M+FA]	2	RP(-) 20ev: 612.3318 --> 552.3098 (100), 327.2321 (55)	662513	143197	570545	159375	0.00320	-0.61	1.37	-1.17
474.4074@12.439606	RP	+	474.4074	12.44				154721	20570	141698	24912	0.00324	-0.57	1.45	-1.10
180.0455@1.563466	HILIC	+	180.0455	1.56				39747	12441	47448	13152	0.00325	0.60	1.57	1.22
681.3246@10.077852	RP	-	681.3246	10.08				63328	10219	56614	11629	0.00325	-0.61	1.38	-1.13
819.5272@12.094173	RP	-	819.5272	12.09				61349	45400	81425	58063	0.00328	0.39	1.10	1.19
1537.1082@12.19372	RP	+	1537.1082	12.19				574049	148675	730467	292972	0.00331	0.71	1.73	1.23
175.0635@5.0259047	RP	+	175.0635	5.03				310988	94292	254553	68622	0.00332	-0.69	1.57	-1.21
755.5468@12.01643	RP	+	755.5468	12.02	PC(34:3)	2	RP(+) 10ev: 756.5564 --> 756.5543 (100), 184.0741 (15)	9087779	4915287	13229119	7554583	0.00332	0.66	1.40	1.48
1537.6108@12.343345	RP	+	1537.6108	12.34				51772	8084	58815	12457	0.00336	0.69	1.53	1.12
160.1207@6.7938137	HILIC	+	160.1207	6.79				42508	30496	61516	42327	0.00340	0.52	1.13	1.46
1541.1387@12.525112	RP	+	1541.1387	12.53				340253	106615	405620	110166	0.00348	0.60	1.54	1.21
813.4974@12.76368	RP	+	813.4974	12.76				75719	18815	79961	30449	0.00348	0.17	0.61	1.12
923.5198@1.120602	HILIC	-	923.5198	1.12				76868	22596	88331	26362	0.00357	0.47	1.25	1.19
241.0717@3.0051851	HILIC	+	241.0717	3.01				24587	9984	34412	20805	0.00364	0.64	1.45	1.32
197.0801@5.6760187	HILIC	+	197.0801	5.68	N-Acetyl-L-Histidine	2	Hilic (+) 10ev: 198.0873 --> 156.0758 (100), 110.0719 (61), 198.0858 (41), 152.0796 (37), 180.0755 (32), 154.1237 (23), 198.1588 (17)	53421	18680	70228	29307	0.00368	0.70	1.53	1.27
494.1644@1.414745	HILIC	-	494.1644	1.41				165019	84946	116575	92392	0.00380	-0.55	1.31	-1.47
286.1935@6.2059126	RP	+	286.1935	6.21	MG(13:1); [M+NH4]+	2	RP(+) 10ev: 304.2274 --> 269.1892 (100), 287.2018 (84), 251.1789 (40), 227.174 (19), 213.164 (18), 241.1942 (18), 199.1507 (16), 90.0575 (14)	330551	145069	250690	129620	0.00385	-0.58	1.37	-1.41
819.593@12.990608	RP	-	819.5930	12.99				289536	85462	330014	102279	0.00397	0.43	1.33	1.16
790.3045@6.2547674	RP	-	790.3045	6.25				193123	73114	151734	84424	0.00406	-0.53	1.34	-1.47

								Control		Preeclampsia		t-test	Cohen's	PLS-DA	fold
Molecular feature	HILIC/RP	Ionization mode	Mass	RT	Identification	ID level	MSMS fragmentation	Mean	SD	Mean	SD	p	d	VIP	change
525.2863@10.108381	RP	+	525.2863	10.11	LysoPE 22:6	2	RP (+) 20ev: 526.2936 --> 44.0486 (100), 385.2730 (63), 62.0592 (48)	1051779	246212	908684	226579	0.00412	-0.61	1.38	-1.16
810.6609@13.676495	RP	+	810.6609	13.68				850683	185486	1023760	306637	0.00414	0.70	1.56	1.18
567.3331@1.1438832	HILIC	+	567.3331	1.14				2572020	709351	2105632	669974	0.00422	-0.68	1.50	-1.22
387.299@0.90089715	HILIC	+	387.2990	0.90				28624	11765	36090	14470	0.00427	0.57	1.36	1.25
73.0527@5.7660303	HILIC	+	73.0527	5.77				81564	29521	102253	35672	0.00431	0.63	1.50	1.25
145.0739@0.78296113	HILIC	+	145.0739	0.78				98839	38232	147259	107862	0.00441	0.66	1.39	1.32
201.1477@3.517562	HILIC	+	201.1477	3.52				74344	62965	114851	70756	0.00445	0.61	1.29	1.75
364.0981@6.453968	HILIC	+	364.0981	6.45				37955	23262	56051	36524	0.00462	0.61	1.38	1.41
380.0196@4.731895	HILIC	-	380.0196	4.73				66456	7721	59749	14108	0.00463	-0.61	1.75	-1.15
399.3347@9.199599	RP	+	399.3347	9.20	Acylcarnitine 16:0	2	RP (+) 20ev: 400.3428 --> 85.0293 (100), 400.3425 (48)	396670	143073	497978	213441	0.00472	0.57	1.35	1.25
1604.0925@12.757357	RP	+	1604.0925	12.76				76212	22441	89246	33157	0.00492	0.47	1.08	1.20
416.224@7.8029394	RP	-	416.2240	7.80				1879864	901434	2763124	1699603	0.00495	0.68	1.64	1.39
982.508@12.569582	RP	+	982.5080	12.57				402881	161582	308062	155432	0.00506	-0.60	1.35	-1.35
525.2852@1.5162116	HILIC	-	525.2852	1.52				79792	23035	66433	20835	0.00507	-0.61	1.41	-1.20
869.5108@12.008849	RP	+	869.5108	12.01				192878	103934	248543	148975	0.00508	0.44	1.00	1.24
609.5327@13.643559	RP	+	609.5327	13.64				79570	37663	106833	61939	0.00516	0.55	1.24	1.32
287.2112@0.85917485	HILIC	+	287.2112	0.86				290571	118670	363767	152414	0.00521	0.54	1.24	1.25
789.552@12.09506	RP	-	789.5520	12.10				88528	22194	112014	50029	0.00524	0.65	1.58	1.21
162.0526@1.8645687	HILIC	-	162.0526	1.86	3-hydroxy-3-methyl-Glutaric acid	2	Hilic (-) 10ev: 161.0454 --> 99.0453 (100), 101.0233 (75), 57.0336 (73), 59.0116 (26)	31885	11600	39058	16355	0.00526	0.51	1.54	1.22
793.5406@12.341885	RP	-	793.5406	12.34				618185	59763	658504	69881	0.00528	0.62	1.37	1.06
486.187@7.764976	RP	+	486.1870	7.76				54134	29722	62166	31000	0.00546	0.26	0.72	1.30
589.447@1.0995506	HILIC	+	589.4470	1.10				60006	55233	58243	41662	0.00551	-0.04	0.44	-1.25
879.6001@12.780818	RP	-	879.6001	12.78	PC(18:0/22:6) [M+FA]	2	RP (-) 20ev: 878.5932 --> 818.567 (100), 878.5894 (35), 327.2354 (23), 283.2746 (6)	3865238	1074286	3242179	1047069	0.00558	-0.59	1.46	-1.21
428.1874@6.9188547	RP	-	428.1874	6.92				1101957	404466	893230	428753	0.00570	-0.50	1.14	-1.29
1696.0623@12.197532	RP	+	1696.0623	12.20				160307	24760	145553	28202	0.00571	-0.56	1.32	-1.11
244.0696@3.490047	HILIC	-	244.0696	3.49				551216	106660	611401	99316	0.00577	0.58	1.39	1.12
956.9792@3.2017598	HILIC	-	956.9792	3.20				58939	38421	39393	24933	0.00580	-0.62	1.56	-1.43
509.349@10.413609	RP	+	509.3490	10.41				308107	97219	235635	104521	0.00584	-0.72	1.57	-1.23
851.6038@10.654809	RP	-	851.6038	10.65				167868	63944	140393	63493	0.00587	-0.43	1.03	-1.27
129.0787@3.85399	HILIC	+	129.0787	3.85				377224	159116	297244	122764	0.00588	-0.57	1.35	-1.30
1020.5793@13.244751	RP	-	1020.5793	13.24				60533	32336	78807	37815	0.00590	0.52	1.14	1.27
270.0854@1.7111067	HILIC	+	270.0854	1.71				31739	11888	40999	17107	0.00591	0.64	1.41	1.28
833.5934@12.78987	RP	+	833.5934	12.79				11725770	3367783	9777456	3186747	0.00592	-0.59	1.42	-1.21
1214.7397@11.987513	RP	+	1214.7397	11.99				415012	129896	327328	122103	0.00596	-0.70	1.48	-1.24
893.5398@11.984011	RP	-	893.5398	11.98				748041	252428	936655	305013	0.00603	0.68	1.47	1.24
512.2976@7.3008566	RP	-	512.2976	7.30				229942	82968	300231	155247	0.00612	0.59	1.50	1.26
191.0614@1.0291665	HILIC	-	191.0614	1.03				41851	10320	47486	13189	0.00623	0.48	1.09	1.14
360.1939@6.5670795	RP	+	360.1939	6.57				351618	125792	289835	156933	0.00634	-0.44	1.23	-1.46
642.5201@14.788431	RP	+	642.5201	14.79				181899	74237	228980	101234	0.00637	0.54	1.24	1.26
400.1561@0.7754666	HILIC	-	400.1561	0.78				108197	87479	72980	63429	0.00652	-0.47	0.97	-1.50
430.3085@9.78305	RP	+	430.3085	9.78				232638	54284	270085	71986	0.00659	0.59	1.36	1.16
923.556@12.826088	RP	+	923.5560	12.83				100626	40361	74559	46178	0.00663	-0.60	1.62	-1.22
734.5575@11.811462	RP	-	734.5575	11.81				169434	26032	190083	39336	0.00666	0.63	1.54	1.11
741.5003@0.9052334	HILIC	-	741.5003	0.91				46245	11331	53737	13366	0.00675	0.61	1.38	1.16
525.2851@1.7353399	HILIC	+	525.2851	1.74				645508	179208	545543	164200	0.00681	-0.58	1.38	-1.19
602.5266@14.800132	RP	+	602.5266	14.80				85778	34570	108044	47635	0.00682	0.54	1.26	1.26
2316.6545@12.346231	RP	+	2316.6545	12.35				244142	44247	282148	70854	0.00687	0.66	1.63	1.14
830.5184@11.696765	RP	-	830.5184	11.70				101063	20942	108771	31588	0.00694	0.29	0.80	1.10
226.0719@4.004437	HILIC	+	226.0719	4.00				117050	20960	105186	18676	0.00717	-0.60	1.28	-1.11
555.3525@10.465272	RP	-	555.3525	10.47	LysoPC(17:0), [M+FA]	2	RP (-) 40ev: 554.3447 --> 269.2488 (100)	196276	96376	163163	61349	0.00729	-0.42	1.03	-1.20
136.0371@5.537356	HILIC	-	136.0371	5.54				242646	58276	281770	73909	0.00737	0.59	1.48	1.16
2334.625@12.342683	RP	+	2334.6250	12.34				270438	44336	309476	75822	0.00738	0.65	1.43	1.13
190.0104@4.7330585	HILIC	-	190.0104	4.73				129187	16445	116082	25949	0.00742	-0.62	1.45	-1.14
202.1428@6.379583	HILIC	+	202.1428	6.38	Putative Symmetric dimethylarginine	3	Hilic (+) 10ev: 203.1500 --> 203.1497 (100), 172.1077 (26), 116.0709 (23), 88.0867 (21), 133.0969 (13), 71.0598 (13), 115.0862 (13), 158.1272 (10)	451683	129727	524147	142421	0.00751	0.53	1.19	1.17
154.0379@3.4898462	HILIC	-	154.0379	3.49				26146	5085	28978	5021	0.00751	0.56	1.31	1.11
893.5383@11.976836	RP	-	893.5383	11.98				672594	322364	894590	358785	0.00753	0.65	1.53	1.46
767.5466@12.898651	RP	-	767.5466	12.90	PE(18:0/20:4)	2	RP (-) 40ev: 766.5355 --> 303.2342 (100), 283.2700 (53), 575.2390 (27)	111900	39541	132974	43266	0.00755	0.51	1.27	1.20
2388.6433@12.2928	RP	+	2388.6433	12.29				374820	94195	276506	157376	0.00769	-0.78	1.76	-1.20
964.4982@11.994775	RP	+	964.4982	11.99				63169	27723	80676	32454	0.00795	0.58	1.32	1.24
525.2854@1.5170193	HILIC	+	525.2854	1.52				82034	23696	69417	24509	0.00805	-0.52	1.23	-1.20
259.0787@3.8940961	HILIC	-	259.0787	3.89				38672	17358	45732	28759	0.00811	0.31	0.86	1.22
515.2913@8.706763	RP	-	515.2913	8.71				261169	196406	409597	364406	0.00820	0.53	1.25	1.52

Molecular feature	HILIC/RP	Ionization mode	Mass	RT	Identification	ID level	MSMS fragmentation	Control		Preeclampsia		t-test	Cohen's	PLS-DA	fold
								Mean	SD	Mean	SD	p	d	VIP	change
921.57@12.386483	RP	-	921.5700	12.39				572230	102879	519944	117402	0.00836	-0.47	1.09	-1.11
691.5513@12.4505205	RP	+	691.5513	12.45				108245	21144	133233	48791	0.00839	0.71	1.69	1.18
871.5557@12.343578	RP	-	871.5557	12.34				1675376	122677	1764173	177386	0.00841	0.59	1.29	1.05
431.304@9.094178	RP	+	431.3040	9.09				105472	57545	141994	108256	0.00844	0.44	0.98	1.39
494.2688@10.141936	RP	-	494.2688	10.14				225492	114534	160188	130174	0.00855	-0.53	1.25	-1.33
887.5127@12.093563	RP	-	887.5127	12.09				41634	42282	67929	50069	0.00858	0.57	1.23	1.38
769.5558@12.535684	RP	-	769.5558	12.54				175146	67252	203131	74571	0.00862	0.39	1.10	1.16
894.3199@1.0983336	HILIC	-	894.3199	1.10				64559	40830	62635	28974	0.00864	-0.06	0.79	-1.32
217.9166@7.745498	HILIC	-	217.9166	7.75				62472	11872	68290	8127	0.00878	0.58	1.31	1.08
159.0894@4.5981483	HILIC	+	159.0894	4.60				216927	260573	323796	301393	0.00883	0.38	0.86	1.90
452.1484@6.2557597	RP	-	452.1484	6.26				136598	32577	118589	38484	0.00894	-0.51	1.34	-1.19
249.0976@1.3908062	HILIC	+	249.0976	1.39				48333	21433	58273	20094	0.00898	0.48	1.24	1.23
442.1192@6.262441	RP	-	442.1192	6.26				63503	9287	57653	14134	0.00912	-0.50	1.38	-1.13
400.1563@5.1728754	RP	-	400.1563	5.17				99085	53921	73975	44812	0.00916	-0.51	1.09	-1.43
559.3361@1.0536345	HILIC	-	559.3361	1.05				27840	16775	24642	13642	0.00917	-0.21	0.82	-1.19
961.5254@11.9844055	RP	-	961.5254	11.98				258206	56069	294836	69117	0.00925	0.59	1.29	1.14
360.1933@6.5544887	RP	-	360.1933	6.55				269171	91665	227448	120095	0.00927	-0.39	1.20	-1.39
872.5032@11.698853	RP	-	872.5032	11.70				94841	40599	99757	53178	0.00936	0.10	0.45	1.12
368.1656@7.537799	RP	-	368.1656	7.54				461408	183471	365566	161282	0.00937	-0.56	1.21	-1.31
315.2406@7.116555	RP	+	315.2406	7.12	Acylcarnitine 10:0	2	RP (+) 20ev: 316.2479 --> 85.0275 (100)	81767	43802	104350	57826	0.00943	0.44	1.03	1.32
217.1315@2.0620322	HILIC	+	217.1315	2.06	Propionylcarnitine	1	Hilic (+) 10ev: 218.1392 --> 218.1392 (100), 85.0286 (72), 159.0654 (21), 60.0808 (13), 144.1017 (5)	578573	330670	833542	578532	0.00970	0.56	1.28	1.30
771.5812@12.986169	RP	-	771.5812	12.99				59474	20168	72877	24235	0.00986	0.60	1.36	1.20
1074.644@1.2260188	HILIC	-	1074.6440	1.23				96205	13346	103993	15294	0.01023	0.54	1.40	1.08
190.0478@3.0819037	HILIC	-	190.0478	3.08				43114	10695	50804	17272	0.01028	0.55	1.24	1.16
637.5647@14.769348	RP	+	637.5647	14.77				81624	33850	101525	45760	0.01039	0.50	1.18	1.25
103.0633@4.707264	HILIC	+	103.0633	4.71	Dimethylglycine	2	Hilic (+) 10ev: 104.0707 --> 58.0648 (100), 104.0715 (12), 45.0332 (9)	78643	36611	96785	40846	0.01067	0.47	1.36	1.24
873.546@12.577486	RP	+	873.5460	12.58				435667	211959	383920	184222	0.01072	-0.26	0.69	-1.28
789.5676@0.9100294	HILIC	-	789.5676	0.91				73442	11782	81664	16163	0.01073	0.59	1.39	1.10
971.4863@1.2032561	HILIC	-	971.4863	1.20				91874	41498	61684	42324	0.01073	-0.72	1.50	-1.37
400.1552@0.93642324	HILIC	-	400.1552	0.94				182903	87118	142661	85030	0.01078	-0.47	1.02	-1.36
525.2852@1.7314906	HILIC	-	525.2852	1.73				515701	155427	433191	141120	0.01080	-0.56	1.31	-1.19
573.3045@10.882501	RP	+	573.3045	10.88				97782	52675	82736	21372	0.01083	-0.41	0.95	-1.15
1565.1353@12.368895	RP	+	1565.1353	12.37				592256	113828	684837	179294	0.01090	0.63	1.55	1.14
875.5614@13.013038	RP	+	875.5614	13.01				367520	81988	426201	111856	0.01105	0.61	1.30	1.15
1585.1274@12.31256	RP	-	1585.1274	12.31				252080	27345	274029	43129	0.01114	0.62	1.71	1.08
552.1242@1.5662707	HILIC	-	552.1242	1.57				35837	15023	23988	15651	0.01118	-0.77	1.68	-1.31
859.4849@12.201837	RP	+	859.4849	12.20				96641	12797	87179	19827	0.01148	-0.58	1.45	-1.09
496.3035@8.823978	RP	-	496.3035	8.82				657047	492094	392057	321273	0.01166	-0.65	1.74	-1.65
132.0245@4.6032333	HILIC	+	132.0245	4.60	L-Methionine	1	Hilic (+) 10ev: 150.0583 --> 104.0521 (100), 133.0337 (92), 102.0547 (65), 56.0492 (64), 61.0107 (43), 150.0577 (27), 74.0224 (22)	163860	42839	190640	57904	0.01172	0.53	1.26	1.16
138.0411@1.0557848	HILIC	+	138.0411	1.06	Urocanic acid	1	Hilic (+) 10ev: 139.0507 --> 139.0522 (100), 121.0378 (37)	85334	57548	59838	44717	0.01175	-0.50	1.14	-1.44
593.2739@10.1039715	RP	-	593.2739	10.10				87882	16187	79642	14642	0.01178	-0.53	1.21	-1.10
989.5584@12.394679	RP	-	989.5584	12.39				190779	59299	161345	60304	0.01178	-0.49	1.09	-1.22
535.3639@1.168041	HILIC	+	535.3639	1.17				117283	49095	100051	39640	0.01208	-0.39	0.91	-1.20
510.283@8.005853	RP	-	510.2830	8.01				464691	239385	662674	271552	0.01220	0.77	2.02	1.21
738.5086@0.8909575	HILIC	+	738.5086	0.89				61734	18439	75934	25376	0.01257	0.65	1.55	1.18
532.265@8.225994	RP	+	532.2650	8.23				106760	40717	133193	52000	0.01265	0.57	1.89	1.23
408.1792@4.006833	HILIC	+	408.1792	4.01				29540	9962	25831	7739	0.01282	-0.42	1.03	-1.17
282.1062@1.0151929	HILIC	-	282.1062	1.02				50755	18668	63446	31381	0.01283	0.51	1.24	1.22
951.5143@12.740202	RP	-	951.5143	12.74				90380	50117	89718	58126	0.01283	-0.01	0.96	1.07
683.1949@12.478571	RP	+	683.1949	12.48				56725	6899	53275	7670	0.01305	-0.47	1.18	-1.07
245.1627@1.2881932	HILIC	+	245.1627	1.29	Isovalerylcarnitine	1	Hilic (+) 10ev: 246.1702 --> 246.1706 (100), 187.0961 (15), 85.0278 (14)	179919	68226	272650	185220	0.01305	0.73	1.50	1.31
506.251@3.834278	HILIC	-	506.2510	3.83				26883	11497	35751	18122	0.01309	0.60	1.35	1.29
147.0892@4.836662	HILIC	+	147.0892	4.84				33177	6177	29806	7257	0.01314	-0.50	1.14	-1.13
659.5443@12.535929	RP	-	659.5443	12.54				184146	96135	239860	120674	0.01384	0.51	1.43	1.26
1026.6403@1.2757983	HILIC	-	1026.6403	1.28				112623	12096	119293	13439	0.01390	0.52	1.20	1.06
577.4123@1.0854855	HILIC	+	577.4123	1.09				60482	14083	52968	11121	0.01410	-0.60	1.48	-1.13
775.5721@11.998684	RP	+	775.5721	12.00				217174	60425	258125	96761	0.01423	0.52	1.34	1.17
400.1556@3.764702	RP	-	400.1556	3.76				131014	76409	99025	65083	0.01424	-0.45	0.97	-1.41
987.525@13.263058	RP	+	987.5250	13.26				73040	26905	77732	32309	0.01429	0.16	0.73	1.13
821.5719@11.986627	RP	-	821.5719	11.99				65325	27603	81181	30671	0.01431	0.54	1.21	1.17
245.1627@2.8372118	RP	+	245.1627	2.84	Isovalerylcarnitine	1	RP (+) 20ev: 246.1697 --> 85.0287 (100)	72192	25260	120597	109398	0.01448	0.72	1.41	1.33
470.2349@8.60907	RP	-	470.2349	8.61				97846	54999	75621	63716	0.01451	-0.37	1.08	-1.33

								Control		Preeclampsia		t-test	Cohen's	PLS-DA	fold
Molecular feature	HILIC/RP	Ionization mode	Mass	RT	Identification	ID level	MSMS fragmentation	Mean	SD	Mean	SD	p	d	VIP	change
454.2388@9.549056	RP	-	454.2388	9.55				740162	712347	529532	243167	0.01480	-0.44	0.96	-1.29
719.5469@12.336857	RP	+	719.5469	12.34				61488	31530	78585	42048	0.01504	0.46	1.23	1.22
581.2809@2.5117543	HILIC	-	581.2809	2.51				42195	18664	50928	21511	0.01519	0.43	1.32	1.23
646.5052@11.2778015	RP	+	646.5052	11.28				80564	15699	90267	20043	0.01526	0.54	1.25	1.12
474.1817@7.8172684	RP	-	474.1817	7.82				74619	20472	87506	24920	0.01558	0.57	1.46	1.17
449.3133@0.9787506	HILIC	-	449.3133	0.98				105835	58783	187884	153238	0.01558	0.77	1.54	1.51
616.5041@14.538459	RP	+	616.5041	14.54				138258	69208	183598	137231	0.01573	0.44	1.03	1.29
851.569@12.186493	RP	-	851.5690	12.19				12548388	1817041	11559908	1818685	0.01590	-0.54	1.32	-1.09
1221.841@11.990557	RP	+	1221.8410	11.99				130747	50210	96744	43957	0.01609	-0.72	1.50	-1.24
430.2024@7.654299	RP	-	430.2024	7.65				108773	58283	136536	65216	0.01612	0.45	1.31	1.27
476.2771@8.012518	RP	+	476.2771	8.01				103929	32861	127215	49935	0.01613	0.56	1.71	1.20
745.5685@12.470499	RP	+	745.5685	12.47				653204	142157	765881	254120	0.01617	0.57	1.54	1.14
920.4742@11.997474	RP	+	920.4742	12.00				301480	90891	317487	132346	0.01622	0.14	0.93	1.16
1569.0483@12.339632	RP	+	1569.0483	12.34				402914	61453	439906	79889	0.01633	0.52	1.32	1.09
195.0534@1.6890193	HILIC	-	195.0534	1.69				38123	18428	52896	40144	0.01634	0.50	1.32	1.31
677.499@11.681348	RP	+	677.4990	11.68				64642	19483	82768	40807	0.01644	0.60	1.55	1.19
765.5352@12.382901	RP	-	765.5352	12.38				84680	43438	103940	59744	0.01657	0.37	0.99	1.15
317.9551@5.4183555	HILIC	-	317.9551	5.42				22860	4288	20863	4287	0.01661	-0.47	1.47	-1.10
791.6218@12.35525	RP	+	791.6218	12.36				765510	234427	628079	230905	0.01704	-0.59	1.43	-1.22
739.5154@12.277361	RP	-	739.5154	12.28	PE(16:0/20:4)	2	RP (-) 40ev: 738.5055 --> 303.2323 (100), 255.2295 (77), 481.5985 (12)	176438	53096	211121	89959	0.01732	0.48	1.19	1.17
593.2722@1.728183	HILIC	-	593.2722	1.73				44047	10175	39124	10043	0.01781	-0.49	1.13	-1.13
833.6143@13.582464	RP	-	833.6143	13.58				1079891	316493	1250242	360850	0.01805	0.50	1.22	1.15
131.0584@5.605106	HILIC	-	131.0584	5.61				27086	8584	30569	8704	0.01809	0.40	0.97	1.14
775.5379@11.868371	RP	-	775.5379	11.87				384867	83417	465652	201736	0.01815	0.57	1.56	1.16
814.3627@1.0971489	HILIC	-	814.3627	1.10				58180	39116	38646	26845	0.01816	-0.59	1.33	-1.41
1132.6707@10.09094	RP	-	1132.6707	10.09				228015	67785	192939	70965	0.01831	-0.51	1.22	-1.24
554.2615@2.5123467	HILIC	-	554.2615	2.51				47559	20185	57172	21117	0.01849	0.47	1.33	1.22
791.5681@12.455627	RP	-	791.5681	12.46				282076	82592	347673	162234	0.01859	0.54	1.40	1.18
267.0952@4.815787	HILIC	-	267.0952	4.82				56295	25613	71429	44274	0.01869	0.43	1.07	1.21
1087.6674@10.09895	RP	+	1087.6674	10.10				559741	257383	460394	218668	0.01869	-0.42	1.09	-1.24
578.2864@12.535063	RP	-	578.2864	12.54				58622	7719	64874	13221	0.01889	0.60	1.79	1.09
143.0949@3.5161455	HILIC	+	143.0949	3.52	Proline betaine	1	Hilic (+) 20ev: 144.1019 --> 144.1026 (100), 84.0804 (30), 58.0658 (27)	12777176	11323868	20049899	13199074	0.01892	0.59	1.28	1.77
117.079@3.724379	HILIC	+	117.0790	3.72	Glycine betaine	1	Hilic (+) 20ev: 235.1654 (2M+H) --> 118.0870 (100), 58.0658 (5), 59.0734 (3)	19002820	3860076	22190270	6781920	0.01911	0.60	1.36	1.14
571.3627@1.1220587	HILIC	+	571.3627	1.12				221743	59268	193296	50593	0.01939	-0.52	1.32	-1.15
285.1368@8.280059	RP	+	285.1368	8.28				191858	183878	123060	101681	0.01970	-0.48	1.37	-1.53
511.2605@8.417275	RP	-	511.2605	8.42				200572	131468	150978	78917	0.02011	-0.47	1.06	-1.25
113.0587@5.557785	HILIC	+	113.0587	5.56				26456	4413	29082	5808	0.02024	0.51	1.20	1.09
216.081@0.95221126	HILIC	-	216.0810	0.95				315116	180357	246560	186503	0.02075	-0.37	1.19	-1.36
430.3084@9.7766285	RP	-	430.3084	9.78				101874	22784	114932	28697	0.02090	0.51	1.16	1.13
633.3253@1.2259898	HILIC	-	633.3253	1.23				47115	5348	50234	7061	0.02096	0.50	1.31	1.06
226.1063@7.120571	HILIC	+	226.1063	7.12				34712	34741	48459	32770	0.02104	0.41	0.97	1.40
543.3332@1.0606511	HILIC	+	543.3332	1.06				2551839	825808	2116594	712057	0.02105	-0.57	1.32	-1.22
280.2403@10.252119	RP	+	280.2403	10.25				207195	74728	164040	100869	0.02121	-0.49	1.21	-1.30
386.1759@5.665372	RP	-	386.1759	5.67				383070	227442	294506	160549	0.02126	-0.46	0.99	-1.38
484.21@7.8236794	RP	-	484.2100	7.82				126896	39892	149150	47441	0.02132	0.51	1.38	1.17
512.2978@8.0061245	RP	-	512.2978	8.01				572153	193490	714389	290045	0.02155	0.59	1.78	1.19
867.4565@11.708105	RP	+	867.4565	11.71				110271	30092	128581	52497	0.02169	0.44	1.18	1.17
496.3029@2.6258078	HILIC	-	496.3029	2.63				111720	152598	132615	133193	0.02170	0.15	0.88	1.44
771.578@12.635234	RP	+	771.5780	12.64	PC(35:2)	2	RP (+) 20ev: 772.5840 --> 772.5840 (100), 184.0727 (94)	330498	186435	402964	217005	0.02175	0.36	0.93	1.22
579.3091@9.994616	RP	-	579.3091	9.99				104894	40762	89051	20754	0.02194	-0.52	1.05	-1.15
1235.8538@11.993524	RP	+	1235.8538	11.99				99078	59984	81520	29861	0.02228	-0.39	0.84	-1.19
845.5705@12.895138	RP	-	845.5705	12.90				778350	93531	731730	97365	0.02239	-0.49	1.11	-1.07
315.241@0.7382813	HILIC	+	315.2410	0.74				163833	81013	203818	96208	0.02239	0.45	1.06	1.25
675.5394@0.9206664	HILIC	+	675.5394	0.92				264597	71568	300816	63335	0.02251	0.54	1.20	1.13
453.1855@11.18902	RP	-	453.1855	11.19				63044	33863	78420	40745	0.02266	0.41	1.45	1.21
779.5472@11.997335	RP	+	779.5472	12.00	PC(16:1/20:4), PC(36:5)	2	RP (+) 20ev: 780.5533 --> 780.5548 (100), 184.0735 (91)	8674133	3908149	11876895	6285075	0.02273	0.63	1.35	1.28
477.322@1.1197767	HILIC	+	477.3220	1.12				32426	7943	28686	5825	0.02289	-0.54	1.30	-1.12
175.1322@3.7250874	HILIC	+	175.1322	3.73				125353	25158	143172	36606	0.02314	0.58	1.32	1.12
563.2409@1.7335339	HILIC	+	563.2409	1.73				40600	11362	35367	11047	0.02348	-0.47	1.10	-1.16
464.2036@3.5655437	HILIC	-	464.2036	3.57				225032	159594	183674	151459	0.02362	-0.27	0.92	-1.49
250.1207@8.708699	RP	+	250.1207	8.71				128906	39913	111663	41425	0.02376	-0.42	1.02	-1.17
280.0461@1.4785385	HILIC	-	280.0461	1.48				111044	39686	95795	38498	0.02403	-0.39	1.17	-1.19
576.5114@14.54596	RP	+	576.5114	14.55				81043	40890	107225	83272	0.02438	0.42	0.99	1.27
188.1273@6.820254	HILIC	+	188.1273	6.82				293114	120662	240867	109066	0.02464	-0.45	1.18	-1.25
601.2051@1.3948046	HILIC	-	601.2051	1.39				33593	19637	46308	26932	0.02470	0.55	1.34	1.35

								Control		Preeclampsia		t-test	Cohen's	PLS-DA	fold
Molecular feature	HILIC/RP	Ionization mode	Mass	RT	Identification	ID level	MSMS fragmentation	Mean	SD	Mean	SD	p	d	VIP	change
729.5306@11.87732	RP	+	729.5306	11.88	PC(32:2)	2	RP (+) 20ev: 730.5377 --> 184.0735 (100), 730.5343 (68)	853369	234654	1075027	624197	0.02479	0.52	1.33	1.18
370.1807@7.301601	RP	-	370.1807	7.30				156970	68977	130632	83205	0.02480	-0.35	0.94	-1.27
949.5349@1.0824422	HILIC	-	949.5349	1.08				234070	50851	256104	45373	0.02481	0.46	1.12	1.10
416.3284@10.466216	RP	-	416.3284	10.47				82856	18007	73609	20166	0.02484	-0.48	1.22	-1.14
681.321@5.476809	RP	+	681.3210	5.48				91713	25779	103510	25596	0.02507	0.46	1.03	1.13
723.522@12.53409	RP	+	723.5220	12.53				87860	38363	101039	45396	0.02517	0.31	0.82	1.12
386.1765@0.8306829	HILIC	-	386.1765	0.83				209382	121008	161947	95389	0.02551	-0.44	0.96	-1.34
400.3342@11.222746	RP	+	400.3342	11.22				571971	541087	417410	418209	0.02553	-0.32	0.92	-1.63
180.0645@2.8150213	RP	+	180.0645	2.82				134911	129374	88456	130522	0.02575	-0.36	0.85	-1.56
182.0787@4.8177304	HILIC	-	182.0787	4.82				1347198	694723	1774755	1427525	0.02618	0.40	0.96	1.23
532.2615@1.4044235	HILIC	-	532.2615	1.40				33080	5345	30709	4889	0.02629	-0.46	1.11	-1.08
467.301@1.2204363	HILIC	+	467.3010	1.22				76386	18543	67643	14312	0.02636	-0.53	1.30	-1.12
423.3344@9.059999	RP	+	423.3344	9.06	Acylcarnitine 18:2	2	RP (+) 20ev: 424.3426 --> 85.0280 (100), 424.3444 (59)	174699	69136	224107	119309	0.02647	0.52	1.28	1.23
500.3893@12.976044	RP	+	500.3893	12.98				120583	20162	111682	25508	0.02659	-0.39	1.17	-1.09
817.5006@12.010333	RP	+	817.5006	12.01				185442	73221	229274	108907	0.02664	0.48	1.16	1.23
272.1143@3.723787	HILIC	+	272.1143	3.72				71461	29915	101479	59922	0.02689	0.67	1.44	1.29
268.1313@8.701549	RP	-	268.1313	8.70				148297	45292	129422	47433	0.02693	-0.41	1.03	-1.17
1053.6566@1.2676214	HILIC	-	1053.6566	1.27				51891	9008	55510	5728	0.02699	0.49	1.11	1.05
219.1107@1.0154812	HILIC	-	219.1107	1.02	Pantothenic acid	1	Hilic (-) 10ev: 218.1037 --> 88.0396 (100), 146.0807 (45), 218.1025 (19)	237817	95365	290529	147820	0.02855	0.43	1.13	1.20
1192.7@0.9425776	HILIC	-	1192.7000	0.94				156751	46803	133927	48089	0.02857	-0.48	1.35	-1.21
474.4068@12.427006	RP	-	474.4068	12.43				131831	17709	123754	19835	0.02860	-0.43	1.32	-1.07
700.5532@0.8998446	HILIC	+	700.5532	0.90				6149126	2069193	6962870	1642391	0.02946	0.44	0.96	1.18
845.5708@12.641301	RP	-	845.5708	12.64				181158	44008	170346	103993	0.02993	-0.15	0.40	1.14
220.0347@4.90566	HILIC	+	220.0347	4.91				38858	11203	45810	20215	0.03000	0.44	1.04	1.14
889.5581@12.993369	RP	-	889.5581	12.99				226806	48626	253901	59856	0.03027	0.50	1.27	1.11
789.5893@13.0940695	RP	-	789.5893	13.09	Plasmenyl-PC 34:1; PC(P-16:0/18:1) [M+FA]	2	RP (-) 40ev: 788.5764 --> 281.2463 (100)	172643	38524	204371	67793	0.03035	0.60	1.53	1.15
129.0422@6.334001	HILIC	+	129.0422	6.33				62404	47475	68146	77968	0.03039	0.09	0.74	1.24
597.2515@10.090644	RP	+	597.2515	10.09				286769	25402	270537	42439	0.03079	-0.48	1.08	-1.08
371.3037@8.699058	RP	+	371.3037	8.70	Acylcarnitine 14:0	2	RP (+) 20ev: 372.3121 --> 85.0288 (100), 372.3130 (26)	79715	46468	93371	39920	0.03117	0.32	1.02	1.23
398.3179@10.486338	RP	+	398.3179	10.49				64762	20447	77596	27919	0.03124	0.53	1.21	1.19
729.5868@0.86800987	HILIC	+	729.5868	0.87				965413	269008	1108108	323338	0.03172	0.48	1.09	1.14
885.5289@12.267553	RP	-	885.5289	12.27				89409	50757	70382	49785	0.03182	-0.38	0.84	-1.32
414.2074@7.454301	RP	-	414.2074	7.45				442693	201473	353413	179872	0.03254	-0.47	1.28	-1.29
2460.646@12.195539	RP	+	2460.6460	12.20				453144	97946	407753	116678	0.03276	-0.42	1.31	-1.13
557.2869@10.120011	RP	+	557.2869	10.12				114498	68640	120525	74014	0.03283	0.08	0.81	1.08
379.2485@9.866395	RP	-	379.2485	9.87				58526	12377	66911	21164	0.03292	0.50	1.13	1.12
464.1845@8.469367	RP	-	464.1845	8.47				170768	61867	144675	60238	0.03350	-0.43	1.24	-1.25
527.3007@10.316618	RP	-	527.3007	10.32				77013	32327	64316	27272	0.03367	-0.43	1.05	-1.21
644.489@0.9676118	HILIC	+	644.4890	0.97				82031	19867	92618	22173	0.03404	0.50	1.13	1.14
823.5854@13.585062	RP	-	823.5854	13.59				142551	42230	153137	63205	0.03429	0.20	0.76	1.15
705.3641@3.108067	HILIC	-	705.3641	3.11				61019	29753	49979	12923	0.03444	-0.52	1.03	-1.15
202.0454@1.4684619	HILIC	-	202.0454	1.47				139319	32214	154135	35107	0.03449	0.44	1.03	1.11
183.0661@1.2741668	HILIC	+	183.0661	1.27				106503	26351	93473	26211	0.03484	-0.50	1.18	-1.11
179.0587@0.96752876	HILIC	-	179.0587	0.97	Hippuric acid	1	Hilic (-) 10ev: 178.0514 --> 178.0510 (100), 77.0393 (76), 134.0606 (65)	551116	460445	780848	725179	0.03498	0.39	0.90	1.38
642.5222@13.789012	RP	+	642.5222	13.79				213720	79279	178576	55310	0.03533	-0.52	1.11	-1.18
856.5153@11.61291	RP	-	856.5153	11.61				56037	10859	60140	16117	0.03544	0.30	0.90	1.09
1014.1732@9.66997	RP	+	1014.1732	9.67				57588	8343	53716	9249	0.03566	-0.44	1.62	-1.08
1044.6198@10.102865	RP	-	1044.6198	10.10				203099	49374	180871	51341	0.03631	-0.44	1.13	-1.13
428.187@1.0038841	HILIC	-	428.1870	1.00				755432	295107	653950	342922	0.03649	-0.32	0.80	-1.23
681.3218@1.2354108	HILIC	+	681.3218	1.24				69062	21424	77830	21094	0.03668	0.41	0.96	1.13
570.2561@3.8983264	HILIC	-	570.2561	3.90				48921	15139	57603	21075	0.03688	0.48	1.41	1.16
726.5677@0.8673108	HILIC	+	726.5677	0.87				1323094	363474	1488097	389980	0.03794	0.44	1.00	1.13
129.0426@6.1003704	HILIC	+	146.0691	6.10	Glutamine	1	Hilic (+) 10ev: 147.0767 --> 130.0508 (100), 84.0450 (90), 147.0771 (12)	3126892	947141	3592597	1019731	0.03798	0.47	1.23	1.19
567.3328@10.086635	RP	+	567.3328	10.09	LysoPC(22:6)	2	RP (+) 20ev: 568.3412 --> 568.3399 (100), 104.1068 (94), 184.0734 (80)	1995253	516856	1744273	512926	0.03803	-0.49	1.16	-1.14
402.1552@10.578373	RP	-	402.1552	10.58				90597	45178	78404	50176	0.03805	-0.26	0.69	-1.23
252.2087@9.446652	RP	+	252.2087	9.45				132977	21947	142147	20920	0.03813	0.43	1.05	1.07
769.5978@12.989144	RP	+	769.5978	12.99	Plasmenyl-PC(36:2); PC(P-36:2)	2	RP (+) 20ev: 770.6055 --> 770.6052 (100), 184.0727 (98)	797120	170744	898470	250917	0.03814	0.48	1.31	1.11
625.2108@10.105425	RP	-	625.2108	10.11				61863	15135	55800	15080	0.03838	-0.40	1.06	-1.12
713.2657@10.087964	RP	-	713.2657	10.09				50727	16526	55499	14147	0.03933	0.31	0.74	1.07
465.3081@2.1922042	HILIC	-	465.3081	2.19				30112	21951	40969	39668	0.03972	0.35	1.05	1.38
569.2481@1.7287089	HILIC	+	569.2481	1.73				25206	5748	22765	5893	0.03978	-0.42	1.01	-1.12
427.3661@9.582857	RP	+	427.3661	9.58				96250	28721	112819	41002	0.04000	0.48	1.21	1.16
334.1013@1.4575667	HILIC	-	334.1013	1.46				528501	152365	615988	200381	0.04055	0.50	1.25	1.16
384.2278@3.2201931	HILIC	-	384.2278	3.22				30111	2988	28762	3446	0.04079	-0.42	1.17	-1.05

								Control		Preeclampsia		t-test	Cohen's	PLS-DA	fold
Molecular feature	HILIC/RP	Ionization mode	Mass	RT	Identification	ID level	MSMS fragmentation	Mean	SD	Mean	SD	p	d	VIP	change
645.4404@10.93041	RP	-	645.4404	10.93				202304	151181	178865	186577	0.04082	-0.14	0.55	-1.38
515.283@12.536035	RP	-	515.2830	12.54				384426	94140	461351	159758	0.04092	0.61	1.73	1.15
2501.6184@12.196392	RP	+	2501.6184	12.20				261900	60777	240042	70617	0.04104	-0.33	1.13	-1.10
633.3257@10.110894	RP	-	633.3257	10.11				263838	28534	276935	30068	0.04123	0.45	1.15	1.05
805.5626@12.192729	RP	+	805.5626	12.19	PC(38:6)	2	RP (+) 20ev: 806.5690 --> 806.5665 (100), 184.0729 (79)	37774739	6090142	35074786	5749044	0.04128	-0.46	1.14	-1.08
539.3288@1.1641488	HILIC	-	539.3288	1.16				41292	28997	41850	32550	0.04138	0.02	0.41	1.15
1048.5522@1.7379898	HILIC	-	1048.5522	1.74				43150	22106	37305	18070	0.04167	-0.29	0.91	-1.22
532.3791@10.712787	RP	+	532.3791	10.71				72257	18686	66412	16638	0.04210	-0.33	0.84	-1.11
575.2884@12.535034	RP	-	575.2884	12.54				163281	32502	166435	66274	0.04216	0.06	1.01	1.09
412.1925@6.762241	RP	-	412.1925	6.76				3310175	1446225	2816616	1797652	0.04243	-0.30	0.95	-1.30
348.2271@10.713488	RP	-	348.2271	10.71				136936	60283	118057	64245	0.04250	-0.30	0.80	-1.20
147.0897@4.7955923	HILIC	-	147.0897	4.80				28422	6695	31820	6999	0.04255	0.50	1.19	1.09
865.5834@12.464234	RP	-	865.5834	12.46				141694	43573	123486	37824	0.04273	-0.45	1.01	-1.14
1050.6418@1.2461473	HILIC	-	1050.6418	1.25				66931	11485	70235	14353	0.04280	0.26	0.78	1.05
416.2221@0.89462477	HILIC	-	416.2221	0.89				209098	111264	169510	113783	0.04296	-0.35	0.79	-1.35
532.2647@4.269912	HILIC	+	532.2647	4.27				88833	39257	105473	39083	0.04299	0.42	1.40	1.19
559.3406@10.542826	RP	-	559.3406	10.54				61650	13035	55762	12050	0.04343	-0.47	1.28	-1.10
414.3133@10.470846	RP	-	414.3133	10.47				107298	23591	118899	25616	0.04354	0.47	1.25	1.11
1378.1993@9.885283	RP	-	1378.1993	9.89				88637	10726	97674	28568	0.04359	0.46	0.98	1.08
720.5177@11.710025	RP	+	720.5177	11.71				514146	217443	615141	243490	0.04359	0.44	1.19	1.10
703.5157@11.760779	RP	+	703.5157	11.76	PC(30:1)	2	RP (+) 20V: 704.5244 --> 184.0729 (100), 704.5226 (62)	265228	74654	332446	189016	0.04373	0.51	1.45	1.17
204.0274@6.0871534	HILIC	-	204.0274	6.09				67951	13683	74584	16166	0.04376	0.44	0.99	1.11
512.2983@3.9010382	HILIC	-	512.2983	3.90				328846	116014	391792	159435	0.04386	0.46	1.43	1.18
272.077@3.9878182	HILIC	-	272.0770	3.99				36282	6241	33548	5119	0.04390	-0.48	1.16	-1.08
83.0369@6.1005697	HILIC	+	83.0369	6.10				50754	16186	58527	15992	0.04406	0.48	1.17	1.15
428.1707@10.714474	RP	-	428.1707	10.71				201810	58151	185848	67437	0.04410	-0.25	0.66	-1.14
749.5232@11.751743	RP	-	749.5232	11.75				140349	42273	164044	88588	0.04414	0.36	1.23	1.13
264.111@3.4249048	RP	+	264.1110	3.42				156218	79988	135977	118517	0.04468	-0.20	1.26	-1.27
486.3744@12.601243	RP	+	486.3744	12.60				393368	51135	371959	66171	0.04474	-0.37	0.98	-1.06
716.5469@1.1019707	HILIC	+	716.5469	1.10				75651	13409	84411	21375	0.04490	0.50	1.41	1.10
161.1163@3.7261555	HILIC	+	161.1163	3.73				44883	6223	48620	9027	0.04522	0.49	1.13	1.07
869.5394@12.066666	RP	-	869.5394	12.07				202182	64926	230131	109561	0.04558	0.32	1.06	1.14
463.276@1.3743294	HILIC	+	463.2760	1.37				98507	64082	132000	92020	0.04565	0.43	1.09	1.31
512.2787@9.833416	RP	-	512.2787	9.83				1125318	885610	767246	722652	0.04605	-0.45	1.10	-1.35
146.0694@6.0891933	HILIC	-	146.0694	6.09	Glutamine	1	Hilic (-) 10ev: 145.0615 --> 145.0623 (100), 127.0513 (99), 109.0407 (38)	1250730	486305	1502159	638227	0.04676	0.45	1.21	1.25
280.2391@10.714261	RP	+	280.2391	10.71				72337	32475	60742	34081	0.04691	-0.35	0.90	-1.19
589.3383@10.092026	RP	-	589.3383	10.09	LysoPC(20:4) [M+FA]	2	RP (-) 40ev: 588.3298 --> 303.2343 (100)	3606293	651863	3328325	656827	0.04700	-0.42	1.05	-1.09
379.2475@9.865471	RP	+	379.2475	9.87				83091	20824	93752	35440	0.04724	0.38	0.92	1.13
280.2409@10.71369	RP	-	280.2409	10.71	FA(18:2)	2	RP (-) 20ev: 279.2333 --> 279.2336 (100)	1420322	589183	1229133	624158	0.04743	-0.32	0.82	-1.19
569.3699@10.542644	RP	-	569.3699	10.54				664426	151649	596803	132282	0.04743	-0.48	1.31	-1.11
661.2675@10.082908	RP	-	661.2675	10.08				107442	11226	102829	11988	0.04766	-0.40	0.99	-1.05
367.1945@10.932657	RP	+	367.1945	10.93				188117	82483	164435	76120	0.04799	-0.30	1.00	-1.15
430.2011@6.905705	RP	-	430.2011	6.91				238178	115636	193498	81769	0.04833	-0.45	1.22	-1.20
821.5248@12.55157	RP	+	821.5248	12.55				393830	172789	463235	128022	0.04842	0.46	1.06	1.12
242.1278@1.8193783	HILIC	+	242.1278	1.82				423054	248564	526925	281648	0.04856	0.39	1.00	1.27
609.5329@13.715334	RP	+	609.5329	13.72				72100	30427	89012	44663	0.04864	0.45	1.11	1.21
296.2142@6.6941433	RP	+	296.2142	6.69				86227	37990	74204	48043	0.04911	-0.28	0.89	-1.27
384.3391@12.567641	RP	+	384.3391	12.57				506390	93574	589276	223257	0.04915	0.52	1.24	1.11
118.0266@1.1084707	HILIC	-	118.0266	1.11	Succinic acid	1	Hilic (-) 10ev: 117.0195 --> 73.0296 (100)	50974	30352	64155	55800	0.04943	0.31	0.84	1.17
358.0618@1.4588267	HILIC	-	358.0618	1.46				130647	35030	116253	43820	0.04995	-0.37	1.07	-1.15

Legend: RT, retention time; ID level, level of identification: 1 = against in-house library, 2 = against public MSMS spectral library, 3 = compound class based on MSMS fragmentation; molecular feature is unknown if identification field is empty.
For t-test the α-level was 0.00065 after correction for multiple testing; molecular features with p-value < 0.00065 are shown with gray background
